# Retinal cell death dependent reactive proliferative gliosis in the mouse retina

**DOI:** 10.1038/s41598-017-09743-8

**Published:** 2017-08-25

**Authors:** Sheik Pran Babu Sardar Pasha, Robert Münch, Patrick Schäfer, Peter Oertel, Alex M. Sykes, Yiqing Zhu, Mike O. Karl

**Affiliations:** 1 TU Dresden, Center for Regenerative Therapies Dresden (CRTD), Fetscherstr, 105, 01307 Dresden, Germany; 2 Deutsches Zentrum für Neurodegenerative Erkrankungen e.V. (DZNE), Arnoldstr, 18, 01307 Dresden, Germany; 3 Max Planck Institute for Molecular Cell Biology and Genetics, Dresden, 01307 Germany

## Abstract

Neurodegeneration is a common starting point of reactive gliosis, which may have beneficial and detrimental consequences. It remains incompletely understood how distinctive pathologies and cell death processes differentially regulate glial responses. Müller glia (MG) in the retina are a prime model: Neurons are regenerated in some species, but in mammals there may be proliferative disorders and scarring. Here, we investigated the relationship between retinal damage and MG proliferation, which are both induced in a reproducible and temporal order in organotypic culture of EGF-treated mouse retina: Hypothermia pretreatment during eye dissection reduced neuronal cell death and MG proliferation; stab wounds increased both. Combined (but not separate) application of defined cell death signaling pathway inhibitors diminished neuronal cell death and maintained MG mitotically quiescent. The level of neuronal cell death determined MG activity, indicated by extracellular signal-regulated kinase (ERK) phosphorylation, and proliferation, both of which were abolished by EGFR inhibition. Our data suggest that retinal cell death, possibly either by programmed apoptosis or necrosis, primes MG to be able to transduce the EGFR–ERK activity required for cell proliferation. These results imply that cell death signaling pathways are potential targets for future therapies to prevent the proliferative gliosis frequently associated with certain neurodegenerative conditions.

## Introduction

Glia cells may have stem-cell-like competence and regenerate neuron loss upon injury and disease of the nervous system in some species, but not in others^1^. One prime example are the radial Müller glia (MG) in the retina that are crucial for the maintenance of visual function and tissue integrity. MG are mitotically quiescent in the healthy mammalian retina, like other glia throughout the CNS. In most types of retinal diseases, mammalian MG undergo major cellular and molecular changes summarized as reactive gliosis, which may have supportive and detrimental effects on neuronal function and survival^2^. Upon retinal injury, MG readily regenerate all retinal cell types in zebrafish, whereas in mammals they do not^3–8^. In humans, MG are a potential source of proliferative disorders and detrimental scar formation that might reduce, cause, or exacerbate neuronal degeneration^9, 10^. Comparative studies of mouse models with different forms of inherited retinal degeneration have indicated that reactive gliosis is highly variable, dependent on disease and mouse strain^11, 12^. Further, various studies have shown limited neuronal regeneration upon experimental stimulation of mammalian MG^13–17^. In zebrafish, HB-EGF stimulation is necessary and sufficient to induce MG-derived neurogenesis^18^. In contrast, both retinal injury, inducing EGFR expression, and HB-EGF or EGF treatment are required to induce cell-cycle re-entry of a small number of MG in rodents^15, 19–23^. EGF treatment stimulates highly limited MG-derived neuronal regeneration *in vivo* ^13^. Recent work has shown that regulated molecular programs control regeneration, and possibly limit it^7, 15, 24^. Retinal-damage-induced mechanisms might differentially trigger, regulate, and restrict glial responses, like regeneration or scarring, which possibly depend on the species and type of disease, and are incompletely understood.

Most recent research has demonstrated that the mechanisms regulating cell death vary between some forms of retinal neurodegenerative diseases and injury^25, 26^. At least three major morphologies of cell death have been described: Apoptotic, necrotic, and autophagic cell death. Retinal detachment has been associated with apoptotic and necrotic photoreceptor death, whereas most mouse models with inherited forms of retinal degeneration involve non-apoptotic cell death^25–29^. However, most retinal pathologies may cause some level of retinal detachment, and the cell death mode upon retinal detachment varies between mouse strains^30^. Various other evidences suggest that cell death mechanisms can be interrelated^31, 32^. For example, previous studies have shown that a pan-caspase inhibitor decreased apoptosis upon retinal detachment, but increased RIPK1-dependent programmed necrosis, whereas a combined blockade efficiently prevented retinal photoreceptor cell loss *in vivo* ^32–34^. Thus, programmed necrosis is an essential and complementary mechanism of photoreceptor death. However, although we know that the type of neuronal cell death may vary between pathologies, the consequences for the entire tissue, as well as for individual cell types such as reactive glia, remain unclear. Work in zebrafish has shown that the amount and type of damage matters, indicating that feedback inhibition of the surviving neurons determines the outcome of the regeneration process^35, 36^. Studies on the genomic response of diseased and injured mammalian retina have revealed that various signaling pathways are involved^37, 38^. However, the complex interactions between dying cells, inflammation, cell loss, and differential glial responses are not completely deciphered.

A major difficulty in addressing the mechanism regulating MG mitotic quiescence and (retinal damage induced) proliferative reactive gliosis in mammals has been the lack of a robust and easily tunable experimental system. In contrast to humans^39–42^ and some animals like rabbits, rodent disease models are rarely associated with profound considerable proliferative gliosis^12, 43, 44^. However, MG reactive gliosis in damaged mouse retina is frequently associated with an activation of cell cycle related regulators^19, 45^. Here, we utilize our recently developed mouse retinal regeneration assay that facilitates studies in an easily controlled environment and recapitulates some major steps of reactive gliosis in mammals *in vivo* and in regenerating zebrafish retina^15, 21, 24^: Organotypic explant culture of juvenile mouse retina induces extensive neuronal cell loss and MG become reactive, involving cell hypertrophy, cell displacement and gliotic hallmark gene expression changes. More than 50% of MG re-enter the cell cycle upon EGF stimulation, some reprogram into a stem cell or neurogenic state, and very few differentiate into neuronal-like cells. The mouse MG proliferative and regenerative response becomes rapidly restricted with increasing mouse age, which could provide a system to identify the processes regulating and limiting regeneration in mammals. Here, we provide evidence suggesting that mouse retinal organotypic culture facilitates studies on the mechanism controlling MG quiescence, and on neuronal cell death dependent MG reactivation and proliferative gliosis. Deciphering the mechanisms governing neurodegeneration-associated glial responses will provide new avenues for therapy development.

## Results

### Spatiotemporal dynamics of cell death and proliferation in retinal explant culture

**Figure 1:**
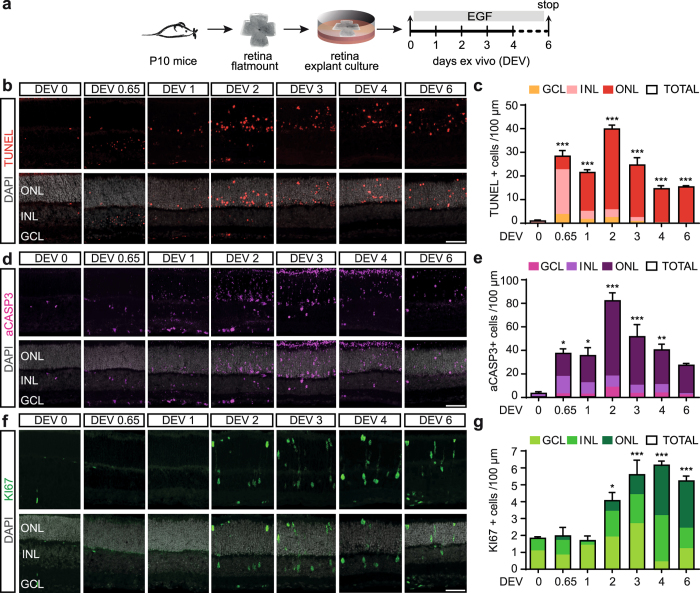
Spatiotemporal cell death and proliferation pattern in retinal explant culture. **(a)** Scheme: Juvenile mouse retinas were dissected at postnatal day 10 (P10) and cultured as organotypic explants with EGF for different periods *ex vivo* and compared to uncultured control (DEV 0). **(b,d,f)** Representative fluorescent images and **(c,e,g)** respective quantitative analysis of retinal sections stained for DNA fragmentation (TUNEL) and active caspase 3 (aCASP3), cycling cells (KI67). Fluorescent images and quantifications were acquired from multiple central retinal regions per independent biological replicate (N). **(b**,**c)** Number of total TUNEL+ cells increased upon retinal explant culture, which occurred first in the INL and GCL at DEV 0.65, followed by the ONL at DEV 2–6 (N ≥ 3 for each timepoint). **(d**,**e)** Temporal analysis showed increased aCASP3+ cell numbers at DEV 0.65 in all retinal layers upon explant culture compared to uncultured control (DEV 0). aCASP3+ peaked at DEV 2 and gradually decreased until DEV 6 (N ≥ 3 for each timepoint). **(f**,**g)** KI67 expression analysis showed rare cell proliferation at DEV 0, which increased upon EGF stimulation in explant culture. Cell proliferation started in the INL at DEV 2 and increased until DEV 4. Bar graph color scheme indicates: GCL, ganglion cell layer; INL, inner nuclear layer; ONL, outer nuclear layer or cell across all layers (total cells). DEV, days *ex vivo*. Data are presented as mean ± SEM, N ≥ 3 (see Table S4). *P < 0.05; **P < 0.01; ***P < 0.001 with 1-way ANOVA (Dunnett’s multiple comparison post hoc test). Scale bars: 50 µm.

We determined the time course of retinal cell death in the previously established mouse retina regeneration *ex vivo* assay^15, 21^ to study the interrelationship with the Müller glia (MG) proliferation response. Postmitotic juvenile retina at postnatal day 10 (P10) were cultured as organotypic explants and treated with EGF (Fig. 1a) to stimulate MG proliferation. To investigate the cell death dynamics in this system, we analyzed DNA fragmentation using the TUNEL assay (Fig. 1b,c) and active (cleaved) caspase-3 (aCASP3) immunostaining (Fig. 1d,e) in tissue sections from cultured retina between days *ex vivo* (DEV) 0.65–6 compared to uncultured control (DEV 0). CASP3 mediates programmed apoptosis and regulates cell death at a relatively early stage. DNA fragmentation occurs in various types of cell death, including programmed apoptosis and necrosis, and is a rather late event of cell death. TUNEL staining detects DNA breaks, a hallmark of apoptosis, but which also occur in necrosis. Data are given as mean with standard error of the mean per 100 µm retinal circumference length. We found that aCASP3+ and TUNEL+ cell nuclei occurred in a time-dependent manner in retina culture (Fig. 1a–e). After 16 hours (DEV 0.65) the majority of TUNEL+ cells (Fig. 1b,c) were seen in the inner nuclear layer (INL), few in the ganglion cell layer (GCL) and outer nuclear layer (ONL). At the same time, the majority of aCASP3+ cells (Fig. 1d,e) started to be found in the INL and ONL and few in the GCL. Cell death transiently increased with ongoing culture time, most strongly affecting photoreceptors (ONL). The total number of TUNEL+ and aCASP3+ cells both peaked at DEV 2 with 40 ± 1 and 82 ± 7 compared to 1 ± 0.3 and 3 ± 0.2 cells in DEV 0 controls, respectively (each timepoint N ≥ 3, both P < 0.001), and declined thereafter (DEV4–6; Fig. 1b–e; for details on biological replicates (N) see Suppl. Table S4). Our data indicate that retinal explant culture induces reproducible cell death in two temporal waves, starting with retinal ganglion cells and interneurons and followed by photoreceptors.

We next investigated the temporal MG proliferation response (Fig. 1f,g) in consecutive sections to the cell death analysis (Fig. 1b–e). We have previously shown and confirmed here (Fig. S2d) that the vast majority of proliferating cells are MG by immunostaining for KI67^15, 24^, an antigen expressed during the active stages of the cell cycle. MG proliferation and nuclear displacement into the ONL started on DEV 2 and further increased until DEV 4 (3 ± 0.4 cells compared to 0 ± 0.000 cells in control, N ≥ 3, Fig. 1g). These results demonstrate that a wave of neuronal death rapidly and extensively builds up within 2 days and precedes a wave of MG proliferation – suggesting that MG proliferation is a result of neuronal death.

### Müller glia cell cycle re-entry depends on retinal damage

**Figure 2:**
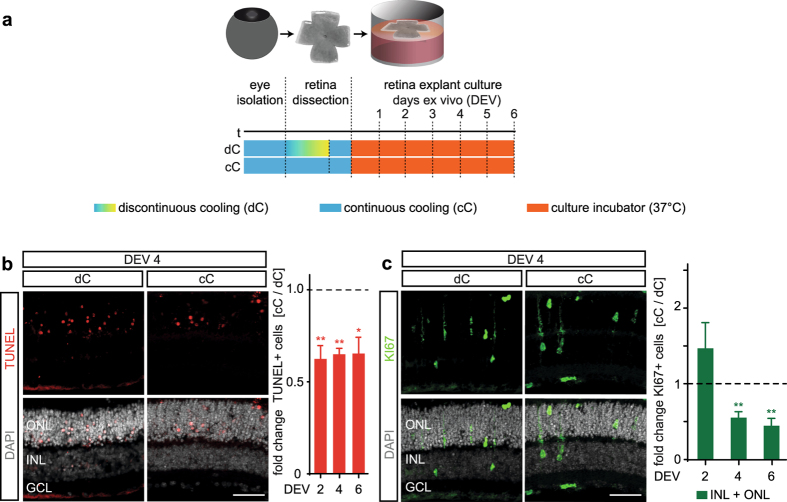
Hypothermia pretreatment reduces neuronal cell death and Müller glia proliferation. **(a)** Scheme: Ordered timed workflow for organotypic retinal explant culture. Both eyes of a mouse were isolated and stored in storage buffer chilled on ice, followed by retinal dissection for flatmounting. Retinas were either maintained in hypothermic conditions (continuous cooled protocol, cC), or were temporarily allowed to rewarm to RT during dissection and returned to hypothermic conditions thereafter (discontinuous cooled protocol, dC). Retinas were cultured at the air-media interface (37 **°**C, 5% CO_2_) and treated with EGF. **(b)** Representative images and quantitative analysis depict that cell death (indicated by TUNEL+ cell nuclei), and **(c)** Müller glia proliferation (indicated by KI67+ cell nuclei) are reduced in cC compared to dC conditions at different days *ex vivo* (cC data was normalized to dC). GCL, ganglion cell layer; INL, inner nuclear layer; and ONL, outer nuclear layer. DEV, days *ex vivo*. Data are presented as mean ± SEM, N ≥ 3 (see Table S4). *P < 0.05; **P < 0.01; ***P < 0.001 with Student’s t-test (unpaired, two-tailed). Scale bars, 50 µm. See Fig. S1.

To demonstrate that damage induced by retinal explant culture is required for a robust MG proliferation response, and to facilitate the application of controlled experimental damage, we developed a protocol to minimize neuronal cell death. We hypothesized that dissecting eyes and retinas under controlled hypothermic conditions (hereafter called the continuous cooling (cC) protocol), applied continuously until retinas are transferred to a cell culture incubator at 37 **°**C (Fig. 2a), might reduce neuronal cell death. According to our original protocol, hereafter referred to as the discontinuous cooling (dC) protocol^15, 21^, the retinas experience a rewarming period during dissection (Fig. 2a). Comparative experiments of retina cultures prepared by the dC and cC protocols revealed that the level of retinal cell death (Figs 2b, S1a) and MG proliferation (Figs 2c, S1b) were both strongly reduced under cC. We found lowered numbers of TUNEL+ cells under cC compared to dC at DEV 2, 4, and 6 (average of all timepoints: 64 ± 3%, N = 12 per variable with N = 4 per timepoint, P < 0.01; Figs 2b, S1a). Relatedly, comparison of MG proliferation at its peak timepoint DEV 4 (and later) showed a two-fold reduction under cC compared to dC (50 ± 16% KI67 cells per ONL and INL, N = 4 per timepoint and variable, P < 0.01; Figs 2c, S1b). Further, the apical nuclear displacement of MG was also reduced under cC conditions (Figs 2c, S1b). In summary, our data show that neuronal cell death and associated MG proliferation in retina culture can be controlled by the dissection procedure. To confirm the latter hypothesis, we used our new modified system (cC conditions) with lower levels of baseline cell death and MG proliferation and investigated whether experimental damage results in increased neuron death and MG proliferation.

**Figure 3:**
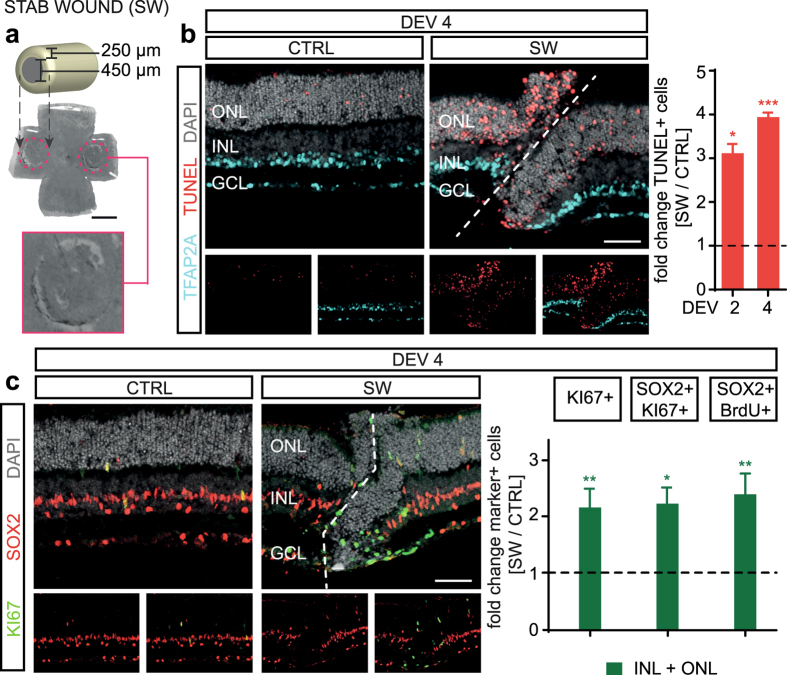
Retinal damage enables EGF-stimulated Müller glia proliferation. **(a)** Retinal stab wound damage was performed by stabbing with a common 200 μl sterile pipette tip through two of four wings of a retinal flatmount causing a circular lesion (see Fig. S2a). Retinas were prepared to achieve a low level of baseline cell death upon explantation and were EGF-treated (cC conditions, see Fig. 2). Top: Drawing of pipette tip dimensions. Middle: Retinal flatmount overview image with dashed lines indicating the stab wound areas that are shown at higher magnification in the lower image. For analysis, each lesion region of interest could be easily identified by a complete interruption and displacement of retinal layers (see Fig. 3b and S2). Two lesion areas of interest (440 µm width) were averaged and compared with internal unharmed control areas (areas >600 µm from the lesion area). **(b)** Cell death was increased upon stab wound injury: Representative image of a retinal lesion area immunostained for cell death (TUNEL), amacrine neurons (TFAP2A), and cell nuclei (DAPI). Quantitative analysis showed a higher number of TUNEL+ cell nuclei in stab wound lesion areas compared to control areas (CTRL). **(c)** Müller glia proliferation was increased in lesion areas compared to control. Image of a retinal lesion area immunostained for the MG marker SOX2 and cell proliferation marker KI67. Quantitative analysis of KI67 as well as SOX2+ KI67+ or SOX2+ BrdU+ double-positive cells indicated increased MG proliferation after stab wound compared to control areas (CTRL). S-phase cell proliferation marker BrdU was given cumulatively throughout explant culture. GCL, ganglion cell layer; INL, inner and ONL, outer nuclear layer. **(b**,**c)** Dashed white lines indicate retinal lesion. DEV, days *ex vivo*. Data are shown as mean ± SEM; N ≥ 3 (see Table S4). *P < 0.05 with Student’s t-test (unpaired, one-tailed). Scale bars: **(a)** scale 1000 µm, **(b**,**c)** 50 µm. Also see Fig. S2.

Controlled physical damage by stab wound injury in defined areas of interest strongly induced neuronal cell death and MG proliferation compared to unharmed control areas within the same retinas (cC conditions). We punctured freshly explanted retinas on the cell culture membrane insert with a sterile 200 µl pipette tip (Figs 3a, S2a), which resulted in a circular lesion in the en face retinal flatmount view, and in an incision that severed all retinal layers in the retinal cross-section view (Figs 3b,c, S2b–c). The lesion areas showed more than three times as many TUNEL+ cells at DEV 2 (N = 3, P < 0.05) and DEV 4 (N = 4; P < 0.001) compared to control areas (Figs 3b, S2b). To specifically monitor MG proliferation, we analyzed co-expression of the MG marker SOX2 with the proliferation marker KI67, and incorporation of the S-phase proliferation marker BrdU, cumulatively applied throughout cultivation (Figs 3c, S2c). Strikingly, there was a two-fold increase in MG proliferation in the lesion areas at DEV 4 (KI67+ 219 ± 31%, N = 4, P < 0.01; KI67+ SOX2+ 228 ± 25% N = 4, P < 0.05; BrdU+ SOX2+ 245 ± 32%, N = 4, P < 0.01) compared to control. In summary, the modified version of our retinal *ex vivo* assay enables reliable and reproducible induction of experimental damage and EGF-dependent MG proliferation. Of note, this system recapitulates previous findings *in vivo*, showing that EGF is not sufficient to induce MG proliferation, but that combined retinal damage and EGF are required^13, 22^. Conditions with low or high levels of neuronal cell death were associated with low and high numbers of MG proliferation, respectively.

### Combined (but not separate) inhibition of distinct cell death signaling pathways is required to reduce neuronal cell death and associated MG proliferation

**Figure 4:**
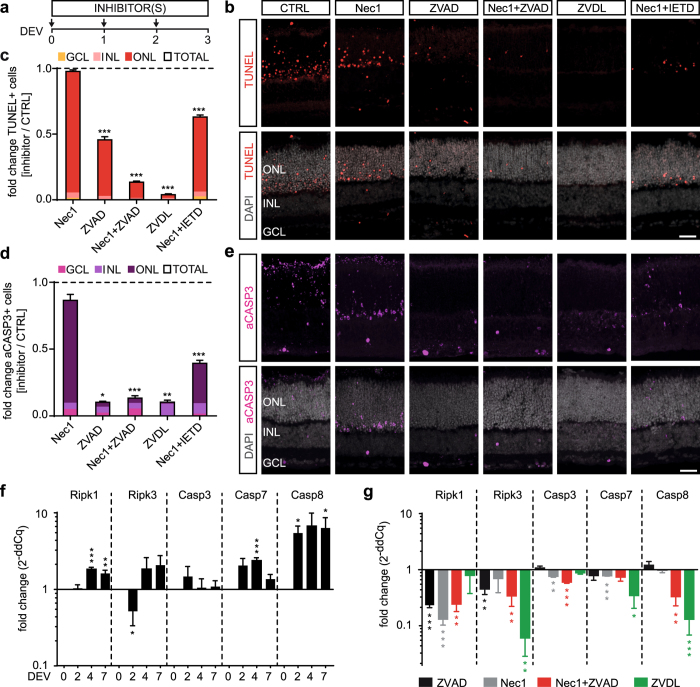
Combined (not separate) cell death pathway inhibition reduces retinal cell death. **(a)** Scheme: EGF-treated retinal explants were cultured until DEV 3 with different cell death pathway inhibitors (Table S1) either separately or in combination, and were compared to solvent controls (CTRL). **(b,e)** Immunofluorescence images and **(c,d)** quantitative analysis of cultured retinal explant sections stained for cell death markers TUNEL and activated caspase 3 (aCASP3) indicated a reduction in cell death depending on the respective inhibitors added: Simultaneous application of necrostatin1 (Nec1) and the pan-caspase inhibitor Z-VAD-FMK (ZVAD) prevent cell death more efficiently than single inhibitors. Nec1 combined with a CASP8 inhibitor (Z-IETD-FMK, IETD) was less efficient than Nec1 with the pan-caspase inhibitor ZVAD (N = 4, each treatment). The ZVDL mixture (ZVAD, Z-DEVD-FMK, and Z-LEHD-FMK) (N = 4) strongly reduced cell death when compared to Nec1+ ZVAD (N = 3). Data are presented as mean ± SEM; N ≥ 3. (**f**,**g)** Gene expression analysis of cell death regulators, receptor-interacting serine/threonine-protein kinases 1 and 3 (Ripk1, Ripk3) and Casp3, Casp7 and Casp8, by QPCR of RNA derived from whole retinal explant samples: **(f)** Temporal analysis indicated gene expression related to apoptotic and necrotic cell death in retinal explants at DEV 2, 4, and 7 compared to uncultured control (DEV 0). **(g)** Gene expression upon cell death inhibitor treatments showed differential expression levels in death pathway genes (N = 4, each inhibitor treatment). Cq values from gene expression data were normalized to the housekeeping gene (Actb) and respective solvent control (ddCq method). All QPCR reactions were run in technical duplicate, N ≥ 3 biological replicates (see Table S4). GCL, ganglion cell layer; INL, inner nuclear layer; ONL, outer nuclear layer; DEV, days *ex vivo*. *P < 0.05; **P < 0.01; ***P < 0.001 with Student’s t-test (unpaired, two-tailed). Scale bars: 50 µm. See Figs S3 and S4.

**Figure 5:**
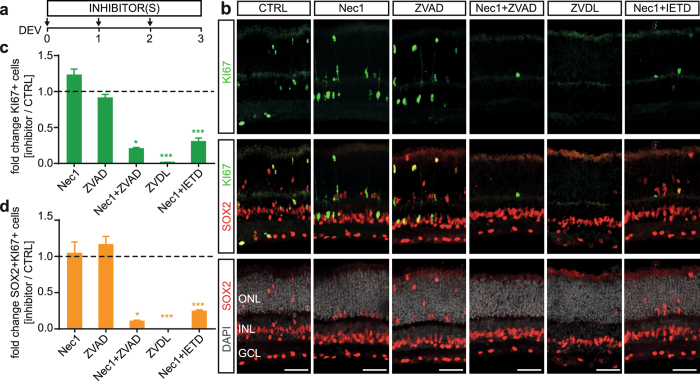
Retinal cell death inhibition prevents Müller glia proliferation. **(a**–**d)** Combined (not separate) death pathway block prevented Müller glia proliferation in EGF-treated retinal explants (data related to Fig. 4). (**a**) Scheme: EGF-treated retinal explants were cultured until DEV 3 with different cell death pathway inhibitors (Table S1) either separately or in combination, and normalized to solvent controls (CTRL). **(b)** Immunofluorescence images and **(c**,**d)** quantitative analysis of retinal sections immunostained for proliferation marker KI67 and MG marker SOX2+ showed that **(c)** total (KI67+) and **(d)** MG (SOX2+ KI67+ double-positive cells) cell proliferation could be inhibited by cell death pathway signaling inhibitors compared to solvent controls. Cell proliferation was almost entirely prevented by combined, but not separate, application of necrostatin1 (Nec1) and pan-caspase inhibitor Z-VAD-FMK (ZVAD). ZVAD reduced cell death (Fig. 4) but not cell proliferation, suggesting compensatory mechanisms. Nec1 combined with a CASP8 inhibitor (Z-IETD-FMK, IETD) also prevented MG proliferation. Compared to Nec1+ ZVAD, the ZVDL mixture (ZVAD, Z-DEVD-FMK, and Z-LEHD-FMK) similarly reduced cell death (Fig. 4) and prevented MG proliferation. GCL, ganglion cell layer; INL, inner nuclear layer; ONL, outer nuclear layer. DEV, days *ex vivo*. Data are presented as mean ± SEM; N = 4 for each inhibitor. *P < 0.05; **P < 0.01; ***P < 0.001 with Student’s t-test (unpaired, two-tailed). Scale bars: 50 µm. See Fig. S5.

To test the hypothesis of neuronal cell death-dependent MG proliferation using a different approach, we sought to reduce cell death using pharmacological inhibitors. Neuronal death has previously been prevented in some retinal disease models, but not others, by applying apoptosis or necrosis signaling pathway inhibitors^28, 32, 46–52^. Thus, we applied selected cell death pathway inhibitors, either separately or in combination, for 3 days in culture (Fig. 4a) and compared them to a solvent control. We determined cell death by TUNEL (Figs 4b,c, S4), aCASP3 immunostaining (Figs 4d,e, S4), and QPCR gene expression analysis (Fig. 4g). MG proliferation was analyzed by co-staining for SOX2 and KI67 on retinal sections (Figs 5, S5). Necrostatin-1 (Nec1) is a potent and selective inhibitor of programmed necrosis that targets receptor-interacting serine/threonine-protein kinase 1 (RIPK1). Nec1 did not affect cell death (N = 4). In contrast, the pan-caspase inhibitor Z-VAD-FMK (ZVAD, perhaps does not inhibit CASP2) significantly reduced the number of aCASP3+ cells in all retinal layers by 90 ± 0.3% (N = 4, P < 0.05) and TUNEL+ cells by 54 ± 2% (N ≥ 3, P < 0.001). MG proliferation remained unchanged in both cases (N = 4, Nec1: KI67+, P = 0.471; KI67+ SOX2+ P = 0.931 and ZVAD: KI67+, P = 0.673; Ki67+ SOX2+ P = 0.628). Inhibition of caspase pathways (ZVAD) upon retinal detachment *in vivo* reduced the number of apoptotic photoreceptors, which was compensated by photoreceptor death via RIPK1-mediated necrosis, whereas Nec1 inhibition reduced necrosis but increased apoptosis. Strikingly, the combined application of apoptosis and necrosis inhibitors efficiently reduced overall photoreceptor loss^32, 34^. Here, we could confirm and extend these findings: Simultaneous application of Nec1 and ZVAD reduced cell death efficiently, with 86 ± 0.2% and 87 ± 1% fewer TUNEL+ and aCASP3 cells (each N ≥ 3, P < 0.001), respectively. This significantly reduced MG proliferation (KI67+ SOX2 + ) by 89 ± 1% (N = 4, P < 0.05, Fig. 5b–d). We also applied a previously reported inhibitor combination (ZVDL) containing ZVAD, Z-DEVD-FMK (blocks CASP9), and Z-LEHD-FMK (CASP3, 7, and 8 inhibitor)^53–56^. ZVDL effectively blocked retinal cell death and MG proliferation: TUNEL+ cells across the entire retina were highly reduced (by 96 ± 1%, N = 4, P < 0.001) and aCASP3 was significantly reduced by 91 ± 1% (N = 4, P < 0.01). MG proliferation was entirely blocked by ZVDL (N = 4, P < 0.001).

To gain insight into the cell death pathways affected by the inhibitors we performed QPCR experiments for major cell death regulators. Temporal gene expression analysis in whole retinal explants at DEV 0, 2, 4 and 7 (Fig. 4f) showed significant upregulation in the range of 1.6-fold to 6-fold for Ripk1 (DEV 4, p < 0.001), Casp7 (DEV 2, p < 0.001) and Casp8 (DEV 2, p < 0.05), an initial transient 2-fold decrease in Ripk3 (DEV 2, p < 0.001), but no change in Casp3. Gene expression analysis after application of cell death pathway inhibitors showed significant changes at DEV 3 compared to the solvent control (Fig. 4g, N ≥ 3): ZVAD (P < 0.001), Nec1 (P < 0.001), and Nec1+ ZVAD (P < 0.01), but not ZVDL, reduced Ripk1 (each N = 4). Ripk3 decreased with ZVAD (P < 0.01), Nec1+ ZVAD (P < 0.01), and ZVDL (P < 0.01). An increase in Ripk3 indicates programmed necrosis in different forms of retinal diseases, including retinal detachment^26, 32, 50, 57^. Nec1 (P < 0.01) and Nec1+ ZVAD (P < 0.001) slightly reduced Casp3, whereas ZVAD and ZVDL had no effect (each N = 4). ZVDL (P < 0.05) reduced Casp7 expression more strongly than Nec1 (P < 0.001, each N = 4). Casp7 expression is observed in some types of retinal degeneration^58^. Interestingly, only Nec1+ ZVAD (P < 0.01) and ZVDL (P < 0.001), but not ZVAD or Nec1, reduced Casp8 (each N = 4). To achieve efficient reduction in neuronal cell death and associated MG proliferation the gene expression analysis suggested that inhibition of Casp8 alone, or in combination with either Ripk1 or Ripk3, might be sufficient: This is achieved using Nec1+ ZVAD and ZVDL, respectively, but not Nec1 or ZVAD alone. Sole inhibition of Ripk1 or Ripk3 does not seem to be effective. Apoptotic cell death can be induced via extrinsic and intrinsic activation, which are mediated by initiators CASP8 and CASP9, respectively. Further, it has been shown that CASP8 acts as a switch by not only inducing apoptotic cell death, but also inhibiting RIPK1-mediated programmed necrosis. Inhibition of CASP8 releases RIPK1 and results in necrosis instead of apoptosis^59^. Following our hypothesis, combined application of Nec1 (RIPK1 inhibitor) and IETD (Z-IETD-fmk, CASP8 inhibitor) significantly reduced the number of TUNEL+ cells (by 37 ± 3%, N = 4, P < 0.001) and aCASP3 (by 61 ± 5%, N = 4, P < 0.001). CASP3 is a known target of CASP8. MG proliferation was strongly reduced by 76 ± 5% upon Nec1+ IETD (KI67+ SOX2+ cells, N = 4, P < 0.001). Our results suggest that cell death might be regulated by apoptotic and non-apoptotic mechanisms in retinal explant culture, which might compensate each other in a similar way to previous findings after *in vivo* retinal detachment^26^. Inhibition of some, but not other, cell death signaling pathways reduced neuronal death and associated MG proliferation, which suggests that not only the amount but also type of cell death might be important.

### Retinal damage and EGFR signaling determine ERK1/2-mediated MG proliferation

**Figure 6:**
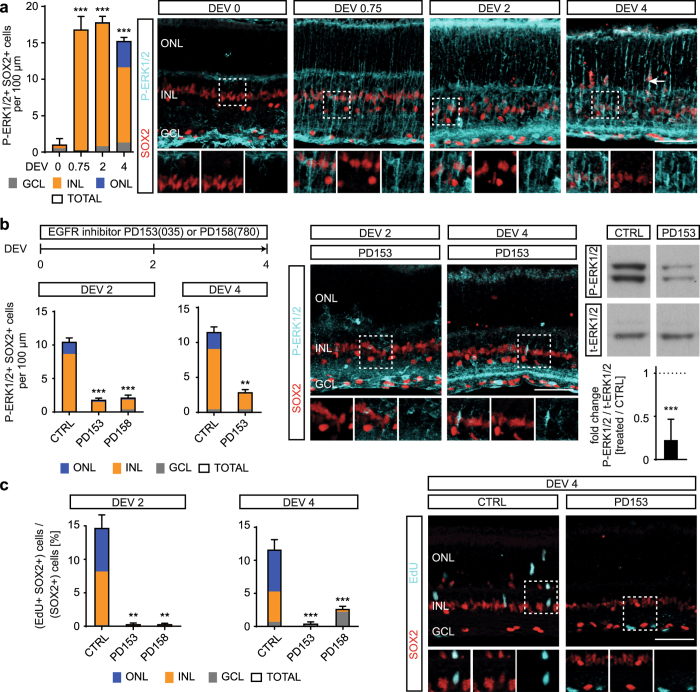
EGFR signaling is necessary for Müller glia proliferation and ERK1/2 activity. **(a)** Potential EGFR activity in Müller glia (MG) in EGF-stimulated retinal explant culture upon indicated treatments and days *ex vivo*. For analysis, retinal sections were immunostained for the MG marker SOX2 and dual phosphorylation (P) of Tyr204 / Y187 of p44/42 mitogen-activated protein kinases 3 and 1 (MAPK3, MAPK1), also called P-ERK1/2 (extracellular signal-regulated kinases 1 and 2). EdU was given cumulatively throughout explant culture to monitor cell proliferation. EdU+ SOX2+ double-positive nuclei indicate MG proliferation. **(a)** Images and quantitative analysis showed that retinal explant culture induced a strong upregulation of P-ERK1/2 immunolabeling with radial morphology. Early on P-ERK1/2 overlaps with SOX2+ MG nuclei located in the INL, and later P-ERK1/2+ SOX2+ MG are also found in the ONL (arrow). Dashed white boxes show regions of interest at higher magnification (DEV 0 & 2, N = 7; DEV 0.75, N = 5; DEV 4, N = 3)). **(b**,**c)** Application of EGFR inhibitors PD153035 or PD158780 throughout retinal explant culture blocked **(b)** P-ERK1/2 upregulation, shown by IHC (N = 4) and Western blot analysis (N = 5), and **(c)** MG proliferation (N = 3) each in comparison to solvent control (CTRL). Bar graph color scheme indicates: GCL, ganglion cell layer; INL, inner nuclear layer; ONL, outer nuclear layer or cell across all layers (total cells). DEV, days *ex vivo*. Data are presented as mean ± SD (Western blot) and ± SEM all others; N ≥ 3 (see Table S4). *P < 0.05; **P < 0.01; ***P < 0.001 with Student’s t-test (unpaired, two-tailed). Scale bars: 50 µm. See Fig. S6.

Retinal injury triggers EGFR upregulation in MG, possibly activating a signaling cascade that may culminate in the phosphorylation of extracellular signal-regulated kinases 1 and 2 (ERK1/2)^19, 20, 57, 60–62^. Here, we investigated whether EGFR and ERK1/2 signaling in mammalian MG possibly transduce some of the observed differential neuronal cell death signaling dependent MG proliferative responses (Figs 2–5). To monitor ERK1/2 activity we performed immunostaining analysis for the MG marker SOX2 and dual phosphorylation (P) of Tyr204/Tyr187 of p44/42 mitogen-activated protein kinases 3 and 1 (MAPK3/1), also called P-ERK1/2. As previously reported in adult mice, we found P-ERK1/2 labeling in the outer and inner plexiform layers, as well as the GCL in undamaged juvenile mouse retinas (Fig. 6a)^60^. Within minutes of retina culture (not shown), P-ERK1/2 transiently increased in MG nuclei and remained at high levels in MG soma and radial processes at DEV 0.75, 2 and 4 (on average: 17 ± 1 P-ERK1/2+ SOX2+ cells /100 µm, dC conditions, N = 15, p < 0.001, Fig. 6a) compared to uncultured control (0.3 ± 0.1, N = 7). With increasing culture time, some P-ERK1/2+ MG became displaced to the ONL and the immunostaining intensity, but not the number of P-ERK1/2+ MG, increased further, suggesting a temporally regulated response.

To find out if EGFR signaling is required for ERK1/2 activation and proliferation of MG in the mouse retina *ex vivo* system, we treated EGF-stimulated retinal explants (dC conditions) with the EGFR inhibitors PD153035 or PD158780 (Fig. 6b,c). Both inhibitors strongly reduced the damage-associated upregulation of P-ERK1/2 in MG by up to 83% (DEV 2: PD153035, N = 5, P < 0.001; PD158780, N = 4, P < 0.001, Fig. 6b, S6). To provide further evidence level for EGFR-dependent ERK1/2 phosphorylation, we performed Western blot analyses on whole retinal explant lysates (Figs 6b, S6c) and found significantly reduced P-ERK1/2 levels after EGFR inhibition (PD153035) on DEV 2 (P-ERK1/2 normalized to total ERK1/2, 23 ± 11%, N = 5, P < 0.001). To monitor MG cell cycle re-entry and proliferation, EdU was applied cumulatively and analyzed by immunostaining. Both EGFR inhibitors led to a complete block in MG cycle re-entry, with up to 98% reduction (total EdU+ SOX2+ cells: DEV 2, N = 5, P < 0.01, PD153035, and PD158780, N ≥ 3, P < 0.01, Fig. 6c). They also prevented damage-induced MG displacement and proliferation (Figs 6c, S6b), but not retinal cell death (Fig. S2a). Our data suggest that EGFR-ERK1/2 signaling mediates the MG activation and proliferation response in the mammalian retina *ex vivo*; this is similar to previous findings *in vivo* ^18–20, 22, 62^.

**Figure 7:**
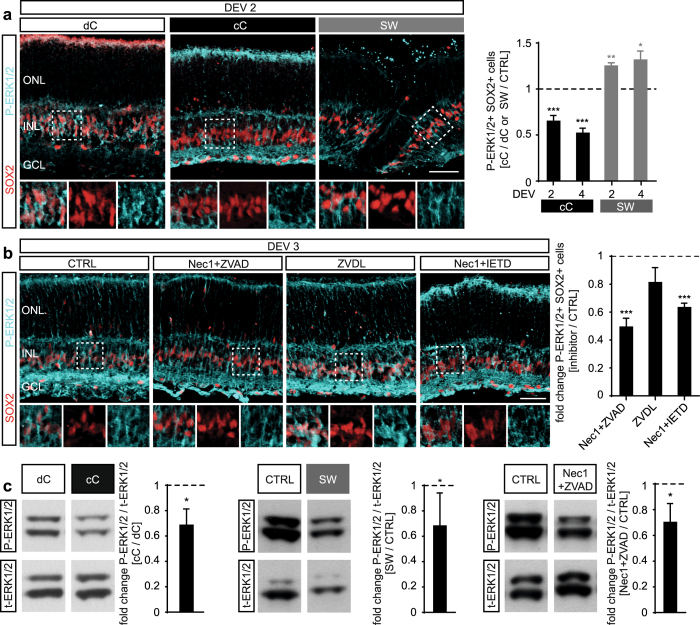
EGF-ERK1/2 activation in Müller glia depends on retinal cell death. Dual-phosphorylation (P) of Tyr204/Y187 of p44/42 mitogen-activated protein kinases p44/42 (MAPK3, MAPK1), also called P-ERK1/2 (extracellular signal-regulated kinases 1 and 2), correlates with experimentally induced cell death in retinal explant culture (related to Figs 2–5): **(a)** P-ERK1/2 expression in Müller glia depends on retinal explant protocol (cC versus dC protocol, see Fig. 2) and experimental retinal damage (stab wound, SW, see Fig. 3, Table S1). Images and quantitative analysis of retinal explant sections immunostained for the MG marker SOX2 and P-ERK1/2 expression showed lower amounts of P-ERK1/2+ MG in retinal explants with reduced baseline cell death (continuously cooled protocol, cC) compared to higher baseline cell death (discontinuously cooled protocol, dC). Stab wound injury (SW) applied to retinal explants with low baseline cell death (cC protocol) increased P-ERK1/2 expression in MG in the lesion area compared to unharmed neighboring control areas ( > 600 µm distant to lesion area; dC vs cC, N = 4; SW, N = 3). **(b)** P-ERK1/2 expression in Müller glia was differentially reduced upon application of cell death pathway inhibitors, which efficiently prevented retinal cell death and MG proliferation (Figs 4–5, N ≥ 3, Tables S1 and S4). **(c)** Western blot analysis of whole retinal explant lysates revealed decreased ERK1/2 activation in experimental paradigms with lower retinal cell death, comparing cC /dC methods and upon cell death inhibition by Nec1+ ZVAD (one data point was removed as an outlier: 2.5-fold different than the mean, P < 0.05 Grubb’s test; complete data shown in Fig. S6c). P-ERK1/2 levels in SW lesioned retinas are lower than in control retinas. GCL, ganglion cell layer; INL, inner nuclear layer; ONL, outer nuclear layer. DEV, days *ex vivo*. Data are presented as mean ± SD (Western blot) and ± SEM all others. *P < 0.05; **P < 0.01; ***P < 0.001 with Student’s t-test (unpaired, two-tailed). Scale bars: 50 µm.

Having determined a role of EGFR-ERK1/2 signaling for MG proliferation in the retina *ex vivo* system, we investigated whether retinal damage differentially controls MG activation and proliferation via EGFR-ERK1/2 signaling. Immunostaining analysis indicated that the number of P-ERK1/2-expressing MG correlates with low (cC) and high (dC and SW) levels of retinal cell death and the associated MG proliferation response (at DEV 4: cC, 54 ± 4%, N = 9, P < 0.001 and SW, 136 ± 10%, N = 4, P < 0.0131; cC normalized to dC and SW normalized to non-lesioned areas of the same cC retina, Fig. 7a). Moreover, reduction of neuronal cell death by defined combinations of cell death pathway inhibitors was associated with decreased numbers of P-ERK1/2-positive MG (Fig. 7b; Nec1+ ZVAD, 51 ± 5%, N = 8, p < 0.001 and Nec1+ IETD, 64 ± 3%, N = 4, p < 0.001; both compared to solvent control). ZVDL showed only a slight, not significant, decrease in ERK1/2-activity (N = 4, P = 0.092). To further support the immunostaining analysis and to determine whether P-ERK1/2 levels correlate quantitatively with the level of retinal damage, we extracted protein from retinal explants of selected experimental paradigms (each N = 5): cC vs dC culture protocol; stab wound and cell death signaling pathway inhibition (Figs 2–5). Expression of P-ERK1/2 and total ERK1/2 (for normalization) were analyzed via Western blot (Figs 7c, S6c): P-ERK1/2 levels were significantly different between retina derived from the two different culture protocols (cC / dC 69 ± 5%, P < 0.05) and upon cell death signaling pathway inhibition (Nec1+ ZVAD / solvent control 71 ± 6%, P < 0.05), confirming the immunostaining data. In retina with defined stab wound lesions, P-ERK1/2 protein levels decreased, in contrast to the slightly increased number of P-ERK1/2-expressing MG in the lesion area (Fig. 7a), but might reflect a lesion-induced reduction of P-ERK1/2 in neuronal cells. A caveat to consider when comparing immunostaining of individual cells and protein expression analysis of whole tissues is the different levels of resolution. Immunostaining showed P-ERK1/2 localization in MG and some other parts of the retina, specifically neurons contributing to the plexiform layers. Our observations support potential differential responses of ERK1/2 in neurons and glia, which is in line with previous studies^60–63^ and further supports the hypothesis of differential reactive (proliferative) gliosis depending on the type of retinal pathology.

## Discussion

**Figure 8:**
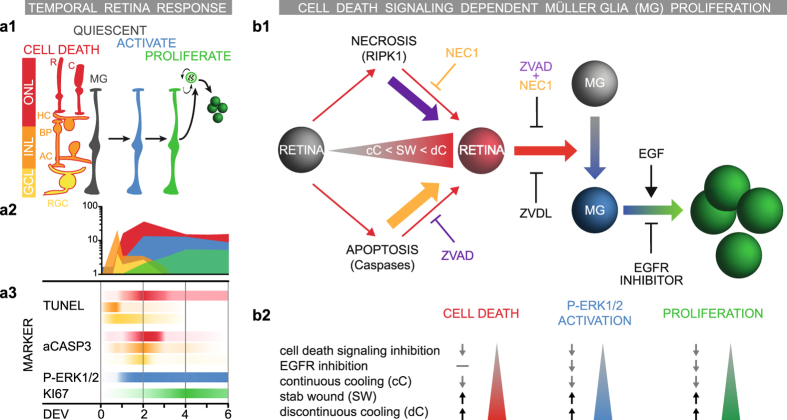
Summary of cell-death-dependent Müller glia (MG) proliferation response in the mouse retina regeneration assay. (**a**) Model of MG response in the retina *ex vivo* assay. **(a1)** MG are quiescent in the postmitotic retina. Retinal explant culture induces neuronal cell death and MG activation. EGF treatment is required for MG proliferation. (ganglion cell layer (GCL, yellow), inner nuclear layer (INL, orange) and outer nuclear layer (ONL, red) **(a2)** Time course of: Retinal cell death (indicated in respective colors shown in **(a1)** based on TUNEL data (dC protocol); MG activation (blue, P-ERK1/2+ SOX2+ data); and MG proliferation (green, SOX2+ KI67+ data). Data are given as mean number of cells per 100 µm central retinal section (log scale). **(a3)** Summary of temporal cell death (aCASP3 and TUNEL) and proliferation (KI67 in ONL+ INL) response (indicated in respective colors shown in **a1**). The color scale ranges from 0 to 100% saturation; data was normalized to the maximum value per variable plotted as 100%. **(b)** Model of retinal cell death and EGFR-ERK-dependent Müller glia proliferation. **(b1)** The discontinuous (dC) and continuous cooling (cC) retinal explant protocols result in either high or low levels of neuronal cell death, respectively. The cC protocol facilitates application of controlled experimental damage, e.g. by stab wound (SW). Cell death levels correlate with the MG activation state, indicated by P-ERK1/2 expression, and the ability of MG to proliferate upon EGF treatment. Combined (not separate) inhibition of defined cell death signaling pathways, RIPK1 (NEC1 inhibitor) and caspases (ZVAD inhibitor), effectively reduced neuronal cell death, MG activation and proliferation. EGF is required, but not sufficient without proper retinal damage, to stimulate MG proliferation, whereas EGFR-inhibition blocked MG proliferation, but not neuronal cell death, suggesting that both are required. The extent of ERK1/2 activation depends on retinal damage and EGFR signaling – correlating with MG activation and proliferation. **(b2)** Overview table of the processes and experimental conditions explained above. Legend: AC, amacrine neurons; BP, bipolar neurons; C, cone photoreceptors; DEV, days *ex vivo*; HC, horizontal neurons; MG, Müller glia; R, rod photoreceptors; RGC, retinal ganglion cells.

The question of how neurons and glia communicate in the diseased nervous system is of considerable interest for maintaining neural vitality and function. Specifically, the functional relationship between diseased (and dying) neurons and glia is incompletely understood. The mouse retina *ex vivo* system offers the possibilities of acute induction of massive neuronal cell death and robust stimulation of MG proliferative gliosis, which cannot yet easily be achieved in the mammalian retina *in vivo*. Further, the *ex vivo* system facilitates the timed application of extrinsic factors, compared to eye injection in animal studies *in vivo*. Of note, photoreceptor detachment may be associated with both apoptotic and non-apoptotic neuronal cell death, whereas inherited retinal diseases mostly involve non-apoptotic cell death^25–30^ suggesting that different modes of neuronal cell death might cause differential gliotic responses. Here, we have established a mammalian retinal *ex vivo* system that facilitates studies on the influence of the amount and type of neuronal cell death on MG reactive gliosis and proliferation. However, to what extent the retinal *ex vivo* system recapitulates pathological processes of *in vivo* models remains incompletely understood. We found that applying continuous hypothermia during eye and retina dissection not only strongly promotes neuron survival^64, 65^, but also prevents MG proliferative gliosis. This indicates a relationship between both processes (Figs 8b, S7). There are various purported mechanisms for tissue hypothermia, like glia-derived neuroprotection, or reduced cell metabolism. Thus, hypothermia might also directly keep MG mitotically quiescent. This modified protocol enabled retinal culture with low levels of cell death and spontaneous MG proliferation, even in the presence of the mitogen EGF. Using this modified system we established an *ex vivo* retinal stab wound model. Stab lesions sufficiently activate ERK1/2 expression in MG of a defined retinal area, which renders them responsive to proliferation in the presence of EGF, whereas areas neighboring the lesion have lower cell death and MG proliferation. In contrast, combined (not separate) inhibition of two major (apoptotic and non-apoptotic) cell death signaling pathways reduced neuronal cell death, ERK1-2 activity and related MG proliferation. ERK1/2 expression in MG depends on both the amount of neuronal cell death and on EGFR signaling, which suggests that the extent of neuronal cell death dependent ERK activation in MG might determine the MG cell cycle re-entry and proliferation response. Our data support previous findings showing that EGFR-ERK signaling induced by retinal damage are required for MG reactive gliosis and proliferation^20, 22, 61–63, 66^. Thus, the mammalian retinal *ex vivo* assay is a powerful system that possibly recapitulates and therefore assists dissecting the mechanisms of neuronal damage-dependent glia cell reactivation and proliferation responses. Further, the retina *ex vivo* system might support the future development of experimental damage and disease models in mouse and human retinal organoid systems^67^ and facilitate studies to decipher the cues differentially regulating reactive proliferative gliosis^1, 2^.

To find out if retinal cell death might be essential for rendering MG responsive to proliferation upon EGF stimulation, we studied the effect of various cell death signaling inhibitors. Apoptosis-dependent compensatory proliferation, i.e. the ability of apoptotic cells to induce proliferation of surviving cells, has been proposed as an evolutionarily conserved mechanism^68^. Given the vital role of caspase activation in apoptotic cell death, blocking their function is a useful strategy for finding out whether apoptosis causes the triggering of reactive proliferative gliosis. Blockage of cell death with pan-caspase or effector-caspase inhibitors impaired tissue regeneration^68–70^ and ameliorated the loss of neuronal cells and function after traumatic brain injury and retinal detachment^48, 71^. Following this approach, we found that a pan-caspase inhibitor reduced the number of cleaved CASP3+ cells in cultured retina, but neither the total amount of cell death nor MG proliferation were affected. Interestingly, different cell death mechanisms, mainly apoptosis, necrosis, and autophagy, might compensate for or switch between each other^31^. It has been shown that programmed necrosis regulated by RIPK1 takes over the control of cell death if caspases are inhibited^59^. Similarly to apoptosis-induced compensatory proliferation, necrotic cells are a source of damage-associated factors that can act as inflammatory mediators. However, to our knowledge necrosis-induced compensatory proliferation has not yet been described. Some studies have shown that application of the RIPK1 inhibitor Nec1 was sufficient to reduce cell death in some, but not other, retinal degeneration models^33^. Here, we observed that combined (but not separate) application of ZVAD and Nec1 effectively prevented neuronal cell death *ex vivo*, as observed *in vivo* ^33^. This was associated with reduced MG cell-cycle re-entry, supporting the hypothesis that cell death is mechanistically linked to the induction of proliferation (Figs 8b, S7). Further, our data suggest that apoptosis is the main mode of cell death in the retina regeneration assay, which might be compensated by necrosis upon apoptosis inhibition. Latter data also suggest that apoptotic and necrotic neuronal cell death each might be sufficient to enable MG proliferative gliosis and inhibition of more than one mode required to prevent it. Following our gene expression data, we hypothesized that inhibition of CASP8, in combination with RIPK1, might be even more specific and therefore similarly effective. Two distinct signaling pathways trigger programmed apoptosis: The intrinsic pathway activated by cellular damage; and the extrinsic pathway induced by the binding of specific pro-apoptotic ligands, like TNF, mediated by the initiator CASP8 activating executioner CASP3. CASP8 has also been shown to negatively regulate necrosis by cleaving RIPK1 and RIPK3^59^. Apoptosis-induced compensatory proliferation has been shown to induce mitogenic cues via initiator or executioner caspases^68, 72^. Inhibition of neuronal cell death and retinal damage dependent MG proliferation was efficient with either ZVDL or Nec1+ ZVAD, which both reduced CASP8 expression and showed a trend increase in RIPK3, a regulator of RIPK1 activation and inhibitor of CASP8-mediated compensatory proliferation in other systems^73^. We found that combined inhibition of CASP8 and RIPK1 reduced cell death less efficiently compared to combined RIPK1 and pan-caspase inhibitors; however, MG proliferation was substantially suppressed by both. This suggests that CASP8-mediated processes might induce MG proliferation, and that other cell death mechanism, which are not priming MG to become proliferation competent, are acting in parallel or are compensating. If so, this would support the hypothesis that cell death, either via CASP8-mediated apoptosis or necrosis, results in the gain of a positive feedback signal from dying cells or loss of negative feedback from surviving cells. This hypothesis is also supported by previous findings^23^ and our data using ZVDL, which has also been shown to reduce adult neurogenesis by decreasing caspase-mediated apoptosis^53, 55, 56^. Initially, it might appear that the ZVDL effect is in conflict with the notion of necrosis compensating for apoptosis. However, the ZVDL mixture contains Z-DEVD-FMK, which was originally identified as a potent CASP3 inhibitor and was later shown to also affect calpain-related necrosis^54^. Calpain-mediated photoreceptor degeneration has been reported^74^, and DEVD-based inhibitors have been shown to rescue retinal degeneration *in vivo* ^75, 76^. This indicates that CASP8, a hub regulator of apoptosis and necrosis, might be part of the mechanistic link of cell-death-dependent MG proliferation. However, chemical cell death inhibitors might also affect non-cell death related processes^77^ and thus could influence cell death and MG proliferation through distinct mechanisms. Further, CASP8 could regulate EGFR-ERK signaling^78^ of MG or affect MG non-autonomously via other cells in the retina, such as vascular endothelia or microglia. It has been suggested that the latter are involved in the MG proliferative response in the retina^79–81^. In summary, not only MG cell intrinsic properties might control reactive gliosis functions and restrict regenerative competence in mammalian retinal pathologies, but also non-autonomous cell extrinsic processes, specifically neuronal cell death.

In our studies, MG activation and cell cycle re-entry upon retina culture is stimulated by EGF, and our data indicate that the level of proliferative activity correlates with the amount of neuronal cell death. Further, EGFR signaling inhibition entirely blocked MG proliferation, which suggests that EGF is required but not sufficient. This data is in agreement with previous studies^13, 15, 19, 20, 22, 61, 62^. In contrast, stimulation of EGFR signaling has been shown to be sufficient and necessary to induce MG proliferation in undamaged zebrafish retina^18^. Thus, differential MG quiescence and activation states might exist between non-regenerative and regenerative species. Mitotic quiescence is generally incompletely understood, and recent data have demonstrated the existence of multiple cell activation states after tissue injury^82, 83^. Several signaling mechanisms have been implicated in regulating reactive gliosis and proliferation in various species^14, 18, 19, 23^, but differential regulation has not yet been completely deciphered. In zebrafish, various single factors are sufficient to induce regeneration^18, 84^, at least some of which are derived from dying neurons^84^. Thus, it will be interesting to determine whether different factors might come into play depending on the type of retinal pathology and species, and if more complex changes are required to trigger proliferative gliosis. Other mechanisms could also regulate the gliotic response, e.g. neuroprotective factors, cell-cell contact, cell debris, neuronal function, inflammation and differential signaling mechanism of MG function^4, 5, 7, 85^ including the EGFR–MAPK-ERK1/2 pathway described here. Recent studies have shown that ERK1/2 activity has an essential function in cell-cycle re-entry regulation and underlies species differences in regenerative competence^86^. Thus, reactive gliosis and glial proliferative disorders in mammals might be a misregulated or limited regenerative program or might be independent entities.

## Methods

### Mouse retinal regeneration assay

For retinal explant culture, eyes from P10 mice were swiftly isolated after euthanasia and directly stored in HBSS supplemented with 0.6% D+ glucose and 5 mM HEPES at pH 7.2 (retinal storage buffer) that had been pre-chilled on ice. Retinal dissection was performed in 2 ml pre-chilled HBSS under a dissection scope either at RT (discontinuously cooled (dC) protocol) allowing the HBSS temperature to increase over time as described previously^15, 20^ or the HBSS was kept on ice during retinal dissection (continuously cooled (cC) protocol). To enable tissue flatmounting for organotypic culture, isolated retinas received four radial cuts at 90° angles and were returned to fresh HBSS chilled on ice. For cultivation, retinas were placed on 6-well tissue culture inserts (Millipore) with the apical side (photoreceptors) facing the membrane in a humidified environment with 5% CO_2_ at 37 °C. Cell culture media (DMEM/F12, US Biologicals, D9807-05) was supplemented (Invitrogen unless stated otherwise) with 1% N2 supplement, 5 mM HEPES (Sigma), 1% penicillin/streptomycin, 1% dialyzed fetal bovine serum, 1 mM L-glutamine, 0.6% D+ glucose, and 0.2% NaHCO_3_. 50% of the medium was changed daily. Recombinant human EGF (50 ng/ml R&D) was supplied daily to stimulate MG proliferation. The S-phase proliferation marker thymidine analog BrdU (10 µM), or EdU (5 µM), were incorporated to monitor cell proliferation. Mice used in this study were C57BL/6JRj (http://www.janvier-labs.com). All experiments were approved by the Landesdirektion Dresden, Germany, and performed in accordance with the TU Dresden and German Federal regulations, German laws, the regulations of the European Union, and the ARVO Statement for the Use of Animals in Ophthalmic and Visual Research.

### Retinal stab wound injury

Retinas were prepared according to our modified protocol to achieve a low baseline level of cell death (cC conditions, see Fig. 2). To induce tissue damage in retinal explants, a 200 µl pipette tip (Biosphere® Filter tip REF 70760211) was stabbed once through the retina, causing a circular lesion. The pipette tip has an inner diameter of 450 µm and a wall thickness of 250 µm (Figs 3a, S2a). Stab wounds were positioned centrally in two opposing wings of the four per retinal explant, and could be readily visualized using brightfield microscopy (Figs 3a, S2a). For analysis, each lesion region could easily be identified by a complete interruption and displacement of retinal layers, which was verified on consecutive retinal cryosections stained with DAPI nuclei stain.

### Drug-based signaling inhibition

For cell death signaling inhibition experiments, the inhibitors listed in Table S1 and their respective DMSO controls were applied daily in full amount at DEV 0, 1 and 2. Inhibitors of EGFR tyrosine kinase activity are listed in Table S1. Inhibitors were applied at the indicated concentrations and respective DMSO controls were added by the daily 50% media exchange.

### Tissue preparation, staining, and imaging analysis

Retinal explants were fixed in 4% PFA at 4 °C overnight, cryoprotected by sucrose, frozen in tissue-freezing medium (Jung), cryosectioned at 20 µm thickness onto superfrost+ slides (Thermo Scientific) and stored at −80 °C. Immunostaining was performed following standard protocols. In brief, blocking solution (10% FBS or 0.5% BSA, 0.3% TritonX-100 in PBS) was applied for 60 min at 37 °C with DNase (AppliChem) before cryosections were incubated with primary antibodies for 1–2 days at 4 °C in staining solution (1% FBS, 0.3% TritonX-100 in PBS), followed by the secondary antibody for 1.5 hours at RT (antibodies information in Table S2). Cell nuclei were labeled using DAPI (Invitrogen). EdU was detected using Click-iT EdU (5-ethynyl-2′-deoxyuridine) Alexa Fluor 488 and 555 kits (Invitrogen) following the manual provided. Cell death analysis by TUNEL (TdT-mediated dUTP-X nick end labeling) staining was performed using the *In Situ* Cell Death Detection Kit Fluorescein and TMR red (Roche). The TUNEL reaction mixture without enzyme solution served as the negative control, and DNase treatment as the positive control. For data analysis, images were acquired from the central region of interest at least 100 µm away from the optic nerve head (center of the retina) and from at least two different central sections per retina. The apical retinal length was measured, and cells were counted using Axiovision Zeiss software tool. Cell counts were normalized to 100 µm retina length. Depending on the experiment, images of at least two central regions of interest were acquired and thereby at least 700 µm total retinal length was analyzed per retina. Images were acquired on the spinning disc Zeiss Axio Observer CXU-X1 or Zeiss Apotome microscopes using Plan-Apochromat 20× objectives. For the retinal overview, tile scan images were acquired on the spinning disc microscope (Plan-Apochromat 10× objective). Optical Z-sectioning was performed at 1 μm intervals for cell quantification and co-localization of double-positive cells. Quantitative analyses were performed on flattened image stacks processed with Zeiss Zen or Zeiss AxioVision, Adobe Photoshop CS5.

### Protein preparation and Western blot analysis

Retinal explants were snap frozen in liquid nitrogen immediately after cultivation, and stored at −80 °C. Individual retina were lysed in 1 × LDS sample buffer (Thermo Scientific) containing 3% b-mercaptoethanol (Sigma), protease inhibitors (cOmplete mini, Roche), and phosphatase inhibitors (PhosSTOP, Roche) on ice for 20 minutes, sonicated, and clarified by centrifugation at 16,000 × *g* for 10 min at 4 °C. Lysates were boiled for 5 minutes and separated on 4–12% NuPage gels, transferred to polyvinylidene fluoride membranes (Millipore), and probed with either mouse anti-ERK1/2 (#4696, Cell Signaling) or mouse anti-phospho ERK1/2 (#5726, Cell Signaling) antibodies. Immunoreactive bands were detected using donkey anti-mouse secondary antibodies (Jackson ImmunoResearch Laboratories) and SuperSignal West Dura Chemiluminescent Substrate (Thermo Scientific), then captured on Amersham Hyperfilm ECL (GE Healthcare). Immunoreactive bands were quantified using Fiji software (https://fiji.sc) and the ratio of P-ERK1/2 to ERK1/2 was determined for individual retinas.

### RNA extraction and QPCR

For RNA extraction, retinas were collected in RLT (Qiagen RNeasy kit). Whole retinas were homogenized with Qiashredder (Qiagen). Total RNA was extracted according to the manufacturer’s instructions (Qiagen RNeasy mini kit). RNA was quantified using a NanoDrop. DNase digestion was performed to prevent contamination from genomic DNA. cDNA synthesis was performed with iScript cDNA synthesis kit (BioRad). Quantitative real-time PCR (QPCR) using SsoFast EvaGreen Supermix (BioRad) was performed on a C1000 Thermal Cycler (BioRad) with the CFX96 Real-time PCR detection system (60 cycles and 60 °C annealing temperature). QPCR reactions were run with at least three biological replicates (N) and as technical duplicates. Actb served as the housekeeping control gene. The primers used for PCR are listed in Table S3.

### Data and statistical analysis

Unless otherwise indicated, data are depicted as mean and standard error of the mean (SEM) and calculated from N ≥ 3 experiments. The numbers of biological replicates are provided in detail in Supplemental Table S4. The probability (P) of the null hypothesis was estimated with Student’s t-test or 1-way ANOVA (Dunnett’s multiple comparison post hoc test) as indicated. Results were considered significant if P < 0.05. GraphPad Prism 7 was used for statistical analyses.

## Electronic supplementary material

Supplementary information

## References

[1] Robel S, Berninger B, Götz M (2011). The stem cell potential of glia: lessons from reactive gliosis. Nat Rev Neurosci.

[2] Bringmann A (2009). Cellular signaling and factors involved in Müller cell gliosis: Neuroprotective and detrimental effects. Progress in Retinal and Eye Research.

[3] Karl MO, Reh TA (2010). Regenerative medicine for retinal diseases: activating the endogenous repair mechanisms. Trends in molecular medicine.

[4] Gorsuch RA, Hyde DR (2014). Regulation of Muller glial dependent neuronal regeneration in the damaged adult zebrafish retina. Exp Eye Res.

[5] Lenkowski JR, Raymond PA (2014). Muller glia: Stem cells for generation and regeneration of retinal neurons in teleost fish. Prog Retin Eye Res.

[6] Jadhav AP, Roesch K, Cepko CL (2009). Development and neurogenic potential of Müller glial cells in the vertebrate retina. Progress in Retinal and Eye Research.

[7] Goldman D (2014). Muller glial cell reprogramming and retina regeneration. Nat Rev Neurosci.

[8] Gallina D, Todd L, Fischer AJ (2014). A comparative analysis of Müller glia-mediated regeneration in the vertebrate retina. Experimental eye research.

[9] Cuenca N (2014). Cellular responses following retinal injuries and therapeutic approaches for neurodegenerative diseases. Progress in Retinal and Eye Research.

[10] Fisher SK, Lewis GP (2003). Müller cell and neuronal remodeling in retinal detachment and reattachment and their potential consequences for visual recovery: a review and reconsideration of recent data. Vision Research.

[11] Suga A, Sadamoto K, Fujii M, Mandai M, Takahashi M (2014). Proliferation Potential of Müller Glia after Retinal Damage Varies between Mouse Strains. PLoS ONE.

[12] Hippert C (2015). Müller Glia Activation in Response to Inherited Retinal Degeneration Is Highly Varied and Disease-Specific. PLoS ONE.

[13] Karl MO (2008). Stimulation of neural regeneration in the mouse retina. Proceedings of the National Academy of Sciences of the United States of America.

[14] Osakada F (2007). Wnt Signaling Promotes Regeneration in the Retina of Adult Mammals. The Journal of Neuroscience.

[15] Löffler K, Schäfer P, Völkner M, Holdt T, Karl MO (2015). Age-dependent Müller glia neurogenic competence in the mouse retina. Glia.

[16] Ueki Y (2015). Transgenic expression of the proneural transcription factor Ascl1 in Müller glia stimulates retinal regeneration in young mice. Proceedings of the National Academy of Sciences of the United States of America.

[17] Yao K (2016). Wnt Regulates Proliferation and Neurogenic Potential of Muller Glial Cells via a Lin28/let-7 miRNA-Dependent Pathway in Adult Mammalian Retinas. Cell Rep.

[18] Wan J, Ramachandran R, Goldman D (2012). HB-EGF is necessary and sufficient for Muller glia dedifferentiation and retina regeneration. Dev Cell.

[19] Close JL, Liu J, Gumuscu B, Reh TA (2006). Epidermal growth factor receptor expression regulates proliferation in the postnatal rat retina. Glia.

[20] Ueki Y, Reh TA (2013). EGF Stimulates Müller Glial Proliferation via a BMP Dependent Mechanism. Glia.

[21] Ueki Y (2012). p53 is required for the developmental restriction in Müller glial proliferation in mouse retina. Glia.

[22] Todd L, Volkov LI, Zelinka C, Squires N, Fischer AJ (2015). Heparin-binding EGF-like growth factor (HB-EGF) stimulates the proliferation of Müller glia-derived progenitor cells in avian and murine retinas. Molecular and Cellular Neuroscience.

[23] Close JL, Gumuscu B, Reh TA (2005). Retinal neurons regulate proliferation of postnatal progenitors and Müller glia in the rat retina via TGFβ signaling. Development.

[24] Schafer P, Karl MO (2017). Prospective purification and characterization of Muller glia in the mouse retina regeneration assay. Glia.

[25] Sancho-Pelluz J (2008). Photoreceptor Cell Death Mechanisms in Inherited Retinal Degeneration. Molecular Neurobiology.

[26] Murakami, Y. *et al*. Photoreceptor cell death and rescue in retinal detachment and degenerations. *Progress in retinal and eye research* **37**, doi:10.1016/j.preteyeres.2013.08.001 (2013). 10.1016/j.preteyeres.2013.08.001 PMC3871865 23994436

[27] Cook B, Lewis GP, Fisher SK, Adler R (1995). Apoptotic photoreceptor degeneration in experimental retinal detachment. Investigative Ophthalmology & Visual Science.

[28] Donovan M, Cotter TG (2002). Caspase-independent photoreceptor apoptosis *in vivo* and differential expression of apoptotic protease activating factor-1 and caspase-3 during retinal development. Cell Death Differ.

[29] Sanges D, Comitato A, Tammaro R, Marigo V (2006). Apoptosis in retinal degeneration involves cross-talk between apoptosis-inducing factor (AIF) and caspase-12 and is blocked by calpain inhibitors. Proceedings of the National Academy of Sciences.

[30] Matsumoto H (2014). Strain Difference in Photoreceptor Cell Death After Retinal Detachment in MiceStrain Difference in Photoreceptor Cell Death. Investigative Ophthalmology & Visual Science.

[31] Vandenabeele P, Vanden Berghe T, Festjens N (2006). Caspase Inhibitors Promote Alternative Cell Death Pathways. Science Signaling.

[32] Trichonas G (2010). Receptor interacting protein kinases mediate retinal detachment-induced photoreceptor necrosis and compensate for inhibition of apoptosis. Proceedings of the National Academy of Sciences of the United States of America.

[33] Murakami Y, Miller JW, Vavvas DG (2011). RIP Kinase-Mediated Necrosis as an Alternative Mechanism of Photoreceptor Death. Oncotarget.

[34] Murakami Y (2012). Receptor interacting protein kinase mediates necrotic cone but not rod cell death in a mouse model of inherited degeneration. Proceedings of the National Academy of Sciences.

[35] Powell C, Cornblath E, Elsaeidi F, Wan J, Goldman D (2016). Zebrafish Müller glia-derived progenitors are multipotent, exhibit proliferative biases and regenerate excess neurons. Scientific Reports.

[36] Sherpa T (2014). Retinal Regeneration is Facilitated by the Presence of Surviving Neurons. Developmental neurobiology.

[37] Rattner A, Nathans J (2005). The genomic response to retinal disease and injury: evidence for endothelin signaling from photoreceptors to glia. J Neurosci.

[38] Roesch K, Stadler MB, Cepko CL (2012). Gene expression changes within Müller glial cells in retinitis pigmentosa. Molecular vision.

[39] Edwards, M. M. *et al*. Idiopathic preretinal glia in aging and age-related macular degeneration. *Experimental Eye Research*, doi:10.1016/j.exer.2015.07.016 (2015). 10.1016/j.exer.2015.07.016 PMC4728061 26220834

[40] Pastor JC (2016). Proliferative vitreoretinopathy: A new concept of disease pathogenesis and practical consequences. Progress in Retinal and Eye Research.

[41] Garweg JG, Tappeiner C, Halberstadt M (2013). Pathophysiology of Proliferative Vitreoretinopathy in Retinal Detachment. Survey of Ophthalmology.

[42] Johnson PT (2003). Drusen-Associated Degeneration in the Retina. Investigative Ophthalmology & Visual Science.

[43] Joly S, Pernet V, Samardzija M, Grimm C (2011). Pax6-positive müller glia cells express cell cycle markers but do not proliferate after photoreceptor injury in the mouse retina. Glia.

[44] Inman DM, Horner PJ (2007). Reactive nonproliferative gliosis predominates in a chronic mouse model of glaucoma. Glia.

[45] Dyer MA, Cepko CL (2000). Control of Muller glial cell proliferation and activation following retinal injury. Nat Neurosci.

[46] Bode C, Wolfrum U (2003). Caspase-3 inhibitor reduces apototic photoreceptor cell death during inherited retinal degeneration in tubby mice. Molecular vision.

[47] Dong K (2012). Necrostatin-1 Protects Photoreceptors from Cell Death and Improves Functional Outcome after Experimental Retinal Detachment. The American Journal of Pathology.

[48] Hisatomi T (2001). Relocalization of Apoptosis-Inducing Factor in Photoreceptor Apoptosis Induced by Retinal Detachment *in Vivo*. The American Journal of Pathology.

[49] Lam TT, Abler AS, Tso MO (1999). Apoptosis and caspases after ischemia-reperfusion injury in rat retina. Investigative Ophthalmology & Visual Science.

[50] Murakami Y (2014). Programmed necrosis, not apoptosis, is a key mediator of cell loss and DAMP-mediated inflammation in dsRNA-induced retinal degeneration. Cell Death and Differentiation.

[51] Perche O, Doly M, Ranchon-Cole I (2007). Caspase-Dependent Apoptosis in Light-Induced Retinal Degeneration. Investigative Ophthalmology & Visual Science.

[52] Rosenbaum DM (2010). Necroptosis, a novel form of caspase-independent cell death, contributes to neuronal damage in a retinal ischemia-reperfusion injury model. Journal of Neuroscience Research.

[53] Ekdahl CT, Mohapel P, Elmér E, Lindvall O (2001). Caspase inhibitors increase short-term survival of progenitor-cell progeny in the adult rat dentate gyrus following status epilepticus. European Journal of Neuroscience.

[54] Knoblach SM (2004). Caspase Inhibitor z-DEVD-fmk Attenuates Calpain and Necrotic Cell Death *in Vitro* and after Traumatic Brain Injury. Journal of Cerebral Blood Flow & Metabolism.

[55] Larson TA, Thatra NM, Lee BH, Brenowitz EA (2014). Reactive Neurogenesis in Response to Naturally Occurring Apoptosis in an Adult Brain. The Journal of Neuroscience.

[56] Thompson CK, Brenowitz EA (2008). Caspase Inhibitor Infusion Protects an Avian Song Control Circuit from Seasonal-Like Neurodegeneration. The Journal of neuroscience: the official journal of the Society for Neuroscience.

[57] Gao S, Andreeva K, Cooper NGF (2014). Ischemia-reperfusion injury of the retina is linked to necroptosis via the ERK1/2-RIP3 pathway. Molecular vision.

[58] Choudhury S, Bhootada Y, Gorbatyuk O, Gorbatyuk M (2013). Caspase-7 ablation modulates UPR, reprograms TRAF2-JNK apoptosis and protects T17M rhodopsin mice from severe retinal degeneration. Cell Death Dis.

[59] Festjens N, Vanden Berghe T, Cornelis S, Vandenabeele P (2007). RIP1, a kinase on the crossroads of a cell’s decision to live or die. Cell Death Differ.

[60] Groeger G, Doonan F, Cotter TG, Donovan M (2012). Reactive oxygen species regulate prosurvival ERK1/2 signaling and bFGF expression in gliosis within the retina. Invest Ophthalmol Vis Sci.

[61] Fischer AJ, Scott MA, Ritchey ER, Sherwood P (2009). Mitogen-activated protein kinase-signaling regulates the ability of Müller glia to proliferate and protect retinal neurons against excitotoxicity. Glia.

[62] Fischer AJ, Scott MA, Tuten W (2009). Mitogen-activated protein kinase-signaling stimulates Müller glia to proliferate in acutely damaged chicken retina. Glia.

[63] Nakazawa T (2008). ERK1 plays a critical protective role against N-methyl-D-aspartate-induced retinal injury. J Neurosci Res.

[64] Reinhard K (2016). Hypothermia Promotes Survival of Ischemic Retinal Ganglion CellsHypothermia Protects Retina During Ischemia. Investigative Ophthalmology & Visual Science.

[65] Schultheiss M (2016). Hypothermia Protects and Prolongs the Tolerance Time of Retinal Ganglion Cells against Ischemia. PLoS ONE.

[66] Wan J, Zhao X-F, Vojtek A, Goldman D (2014). Retinal injury, growth factors and cytokines converge on β-catenin and pStat3 signaling to stimulate retina regeneration. Cell reports.

[67] Völkner M (2016). Retinal Organoids from Pluripotent Stem Cells Efficiently Recapitulate Retinogenesis. Stem Cell Reports.

[68] Ryoo HD, Bergmann A (2012). The Role of Apoptosis-Induced Proliferation for Regeneration and Cancer. Cold Spring Harbor perspectives in biology.

[69] Fan Y, Bergmann A (2008). Apoptosis-induced compensatory proliferation. The Cell is dead. Long live the Cell!. Trends Cell Biol.

[70] Li F (2010). Apoptotic Cells Activate the “Phoenix Rising” Pathway to Promote Wound Healing and Tissue Regeneration. Science signaling.

[71] Zacks DN, Hänninen V, Pantcheva M, Grosskreutz C, Miller JW (2003). Caspase Activation in an Experimental Model of Retinal Detachment. Investigative Ophthalmology & Visual Science.

[72] Bergmann A, Steller H (2010). Apoptosis, stem cells, and tissue regeneration. Sci Signal.

[73] Vucur M (2013). RIP3 inhibits inflammatory hepatocarcinogenesis but promotes cholestasis by controlling caspase-8- and JNK-dependent compensatory cell proliferation. Cell Rep.

[74] Paquet-Durand F (2006). Calpain is activated in degenerating photoreceptors in the rd1 mouse. J Neurochem.

[75] Sánchez-Migallón MC, Valiente-Soriano FJ, Nadal-Nicolás FM, Vidal-Sanz M, Agudo-Barriuso M (2016). Apoptotic Retinal Ganglion Cell Death After Optic Nerve Transection or Crush in Mice: Delayed RGC Loss With BDNF or a Caspase 3 InhibitorRGC Loss and Caspase 3 Activation After Axotomy in Mice. Investigative Ophthalmology & Visual Science.

[76] Yoshizawa K (2000). Caspase-3 Inhibitor Rescues N -Methyl- N -nitrosourea-induced Retinal Degeneration in Sprague–Dawley Rats. Experimental Eye Research.

[77] Nakajima YI, Kuranaga E (2017). Caspase-dependent non-apoptotic processes in development. Cell Death Differ.

[78] Finlay D, Howes A, Vuori K (2009). Critical Role for Caspase-8 in EGF Signaling. Cancer research.

[79] White DT (2017). Immunomodulation-accelerated neuronal regeneration following selective rod photoreceptor cell ablation in the zebrafish retina. Proc Natl Acad Sci USA.

[80] Fischer AJ, Zelinka C, Gallina D, Scott MA, Todd L (2014). Reactive microglia and macrophage facilitate the formation of Muller glia-derived retinal progenitors. Glia.

[81] Todd L, Squires N, Suarez L, Fischer AJ (2016). Jak/Stat signaling regulates the proliferation and neurogenic potential of Muller glia-derived progenitor cells in the avian retina. Sci Rep.

[82] Cheung, T. H. & Rando, T. A. Molecular regulation of stem cell quiescence. *Nature reviews. Molecular cell biology* **14**, doi:10.1038/nrm3591 (2013). 10.1038/nrm3591 PMC3808888 23698583

[83] Rodgers JT (2014). mTORC1 controls the adaptive transition of quiescent stem cells from G(0) to G(Alert). Nature.

[84] Nelson CM (2013). Tumor necrosis factor-alpha is produced by dying retinal neurons and is required for Müller glia proliferation during zebrafish retinal regeneration. The Journal of neuroscience: the official journal of the Society for Neuroscience.

[85] Hollborn M (2005). Changes in retinal gene expression in proliferative vitreoretinopathy: glial cell expression of HB-EGF. Molecular vision.

[86] Yun MH, Gates, Phillip B, Brockes (2014). Jeremy P. Sustained ERK Activation Underlies Reprogramming in Regeneration-Competent Salamander Cells and Distinguishes Them from Their Mammalian Counterparts. Stem Cell Reports.

