# Supplementary material for: Retinal cell death dependent reactive proliferative gliosis in the mouse retina
Source: Sci Rep. 2017 Aug 25;7:9517. doi: 10.1038/s41598-017-09743-8 (PMC5572737; doi:10.1038/s41598-017-09743-8)
Supplement: Supplementary file 1 — Supplementary information [file 41598_2017_9743_MOESM1_ESM.pdf]

Retinal cell death dependent reactive proliferative gliosis in the mouse retina.

Sheik Pran Babu Sardar Pasha, Robert Münch, Patrick Schäfer, Peter Oertel, Alex M. Sykes, Yiqing Zhu & Mike O. Karl

SUPPLEMENTAL FIGURE LEGENDS:

SUPPLEMENT FIGURE S1: Supplement data for hypothermia pretreatment reduces neuronal cell death and Müller glia proliferation. Related to Fig.2. **(a)** Hypothermic pretreatment by application of continuous cooling (cC) compared to discontinuous cooling (dC) protocols (see Fig.2) significantly reduced amount of TUNEL+ cells throughout retina explant cultivation analyzed at day ex vivo (DEV) 2, 4 and 6 (dataset related to Fig.2b). Overall retinal thickness was not affected. **(b)** In cC compared to dC conditions lower numbers of MG re-entered the cell cycle indicated by analysis of KI67+ cells; and fewer number of KI67+ cells appeared in the outer nuclear layer indicating reduced MG displacement (dataset related to Fig.2c). KI67+ cells in the GCL are not MG shown by absence of SOX2 costain. Bar graph color scheme indicates data of total cells, GCL, ganglion cell layer; INL, inner nuclear layer; ONL, outer nuclear layer. Data are represented as mean ±SEM. See Table S4 for number of biological replicates (N). Statistics were computed using Student's t-test, unpaired and two-tailed. Significant values were represented as * $P<0.05$; ** $P<0.01$; *** $P<0.001$. Scale bars: 50µm.

SUPPLEMENT FIGURE S2: Supplement data for time course analysis of cell death and proliferation in retinal SW injury. Related to Fig.3. **(a)** Scheme stab wound (SW) application and analysis: Acutely explanted retina (cC condition) were stabbed in two opposing areas with a sterile 200 µl pipette tip and cultured for defined days ex vivo (DEV). Retinas were subsequently processed for cryosectioning and immunostaining. Quantitative analysis was performed on images of a 440 µm wide region of interest (ROI) across the stab wound injured area on immunostained central retinal sections. Internal control areas (CTRL) were chosen randomly at least 600 µm away from SW region of interest. Two SW and CTRL ROI were averaged per biological replicate (N). **(b)** Images and graph show that in the SW areas the amount of TUNEL+ cells at DEV 2 and DEV 4 increased (dataset related to Fig.3b). **(c)** SW areas with increased cell death were associated with higher rates of MG proliferation, confirmed by SOX2+ MG incorporating BrdU (cumulatively applied throughout cultivation) and by KI67+SOX2+ costaining at DEV 4: shown by images and quantitative analysis (dataset related to Fig.3c). **(d)** Analysis of MG markers SOX2 and SOX9 in the retina ex vivo system supports findings in previous reports [1-4]: Images and quantitative analysis of proliferating (BrdU+) cells colabeled for

SOX2+ and SOX9+ in cultured retina explants on day ex vivo 4 (DEV) indicates similar numbers of proliferating MG. Retinal stab wounds (SW) increase MG proliferation compared to unlesioned control based on SOX2 and SOX9 staining analysis. As previously reported some SOX2 also labels some astrocytes in the GCL and a subset of amacrines in the INL, and some SOX9 cells are also astrocytes in the GCL. GCL, ganglion cell layer; INL, inner nuclear layer; ONL, outer nuclear layer. Data are represented as mean ±SEM. See Table S4 for number of biological replicates (N). Statistics were computed using Student's t-test, unpaired and two-tailed. Significant values were represented as * P<0.05; ** P<0.01; *** P<0.001. Scale bars: 50 µm.

SUPPLEMENT FIGURE S3: Supplement data for combined (not single) death pathway inhibition reduces retinal cell death. Datasets are related to Fig.4A-E. Graph depicting quantitative analysis of **(a)** DNA fragmentation (TUNEL+) and **(b)** cell apoptosis (aCASP3+) per 100 µm immunostained retinal section that is shown as fold change in Fig.4A-E. GCL, ganglion cell layer; INL, inner nuclear layer; ONL, outer nuclear layer. Data are presented as mean ±SEM. See Table S4 for number of biological replicates (N). Statistics were computed using Student's t-test, unpaired and two-tailed. Significant values were represented as * P<0.05; ** P<0.01; *** P<0.001. Scale bars: 50 µm.

SUPPLEMENT FIGURE S4: Overviews images of TUNEL and aCASP3 stained retinal sections from the cell death pathway inhibition experiments. Datasets are related to Fig.4. EGF-treated retinal explants were cultured with different cell death pathway inhibitors (Table S1) either separately or in combination compared to solvent controls (CTRL) until day ex vivo 3. Exemplary confocal overview images of retinal sections immunostained for cell death markers **(a)** TUNEL and **(b)** aCASP3. Scale bars: 1 mm.

SUPPLEMENT FIGURE S5: Supplement data for retinal cell death inhibition prevents Müller glia proliferation. Datasets are related to Fig.5c-d. Quantitative analysis of retinal sections immunostained for proliferation marker KI67 and MG marker SOX2+ showed retinal layer wise that **(a)** cell proliferation (KI67+) and **(b)** MG proliferation (SOX2 KI67 double-positive cells) could be inhibited by cell death pathway signaling inhibitors compared to solvent controls. Graphs depict data per 100 µm immunostained retinal section. GCL, ganglion cell layer; INL, inner nuclear layer; ONL, outer nuclear layer. Data are presented as mean ±SEM. See Table S4 for number of biological replicates (N). * P<0.05; ** P<0.01; *** P<0.001 with Student's t-test (unpaired, two-tailed). Scale bars: 50 µm.

SUPPLEMENT FIGURE S6: Supplement data for EGF-R inhibition prevents Müller glia cell cycle re-entry cell death-independently. Datasets are related to Fig.6. **(a)** EGFR inhibition with small molecule inhibitors PD153035 and PD158780 (500 nM) had no influence on cell death suggested by quantitative analysis of TUNEL staining and retinal layer thickness. **(b)** Images and graphs depicting that EGFR inhibition led to significantly lower number of SOX2+ cells per 100 µm retina section after DEV 2 and DEV 4 supporting the decreased MG proliferation shown in Fig.6. **(c)** Protein extracts from whole retinal explants cultured for indicated days ex vivo (DEV) were used for Western blot analysis of ERK1/2 activation assessed by quantification of phosphorylated (P) P-ERK1/2 versus t-ERK1/2 (total ERK1/2). Data for all biological replicates (N=5) are shown, referring to Fig.6b and Fig.7c. In accordance to the immunostaining analysis, levels of P-ERK1/2 were decreased upon (top left) inhibition of EGFR signaling, (top right) hypothermic pretreatment in continuously cooled (cC) compared to discontinuously cooled (dC) conditions and (bottom left) upon cell death signaling inhibition by Nec1+ZVAD. ERK1/2 activity did not increase in retina with stab wound compared to unlesioned controls. Data are represented as mean ±SEM. See Table S4 for number of biological replicates (N). Statistics were computed using Student's t-test, unpaired and two- tailed. Significant values were represented as * $P<0.05$; ** $P<0.01$; *** $P<0.001$. GCL: ganglion cell layer, INL: inner nuclear layer, ONL: outer nuclear layer. Scale bars: 50 µm

SUPPLEMENT FIGURE S7: Supplement figure for summary of cell death–dependent Müller glia proliferation response in the mouse retina regeneration assay. The mouse retina ex vivo assay allows controlled cultivation of juvenile retinas at postnatal day 10 (P10) for at least 6 days ex vivo (DEV) and cell cycle re-entry and proliferation of formerly postmitotic Müller glia (MG), when treated with epidermal growth factor (EGF). Retina explant culture leads to neuronal cell death, which can be significantly reduced by application of continuous hypothermia during eye and retina dissection (continuous cooling protocol, cC). Neuronal cell death in retina explants can be increased via interruption of continuous hypothermia (discontinuous cooling protocol, dC), or via application of controlled mechanical stab wound injury (SW). SW was performed by puncturing two opposing retinal explant leaves with an 200 µl pipette tip. Increased retinal cell death levels caused increased P-ERK1/2 activation levels in MG and MG cell cycle re-entry. MG reactivation and proliferation can be completely inhibited by EGF-R inhibition. Interestingly, combined, but not single, application of of apoptosis and necrosis signaling pathway inhibitors significantly decreased MG reactivation and proliferation. In sum, our data suggests that MG compensatory proliferation requires (i) retinal cell death and (ii) stimulation of EGF-R signaling dependent downstream activation of ERK-1/2. Our results support the hypothesis that cues from surviving or dying cells, possibly by either necrosis or apoptosis, may prime an activation state of MG that renders them competent to respond with cell proliferation upon EGF-stimulation.

Supplemental References

1 Fischer, A. J., Zelinka, C. & Scott, M. A. Heterogeneity of glia in the retina and optic nerve of birds and mammals. *PLoS One* **5**, e10774, doi:10.1371/journal.pone.0010774 (2010).

2 Karl, M. O. *et al.* Stimulation of neural regeneration in the mouse retina. *Proceedings of the National Academy of Sciences of the United States of America* **105**, 19508-19513, doi:10.1073/pnas.0807453105 (2008).

3 Löffler, K., Schäfer, P., Völkner, M., Holdt, T. & Karl, M. O. Age-dependent Müller glia neurogenic competence in the mouse retina. *Glia* **63**, 1809-1824, doi:10.1002/glia.22846 (2015).

4 Schafer, P. & Karl, M. O. Prospective purification and characterization of Muller glia in the mouse retina regeneration assay. *Glia* **65**, 828-847, doi:10.1002/glia.23130 (2017).

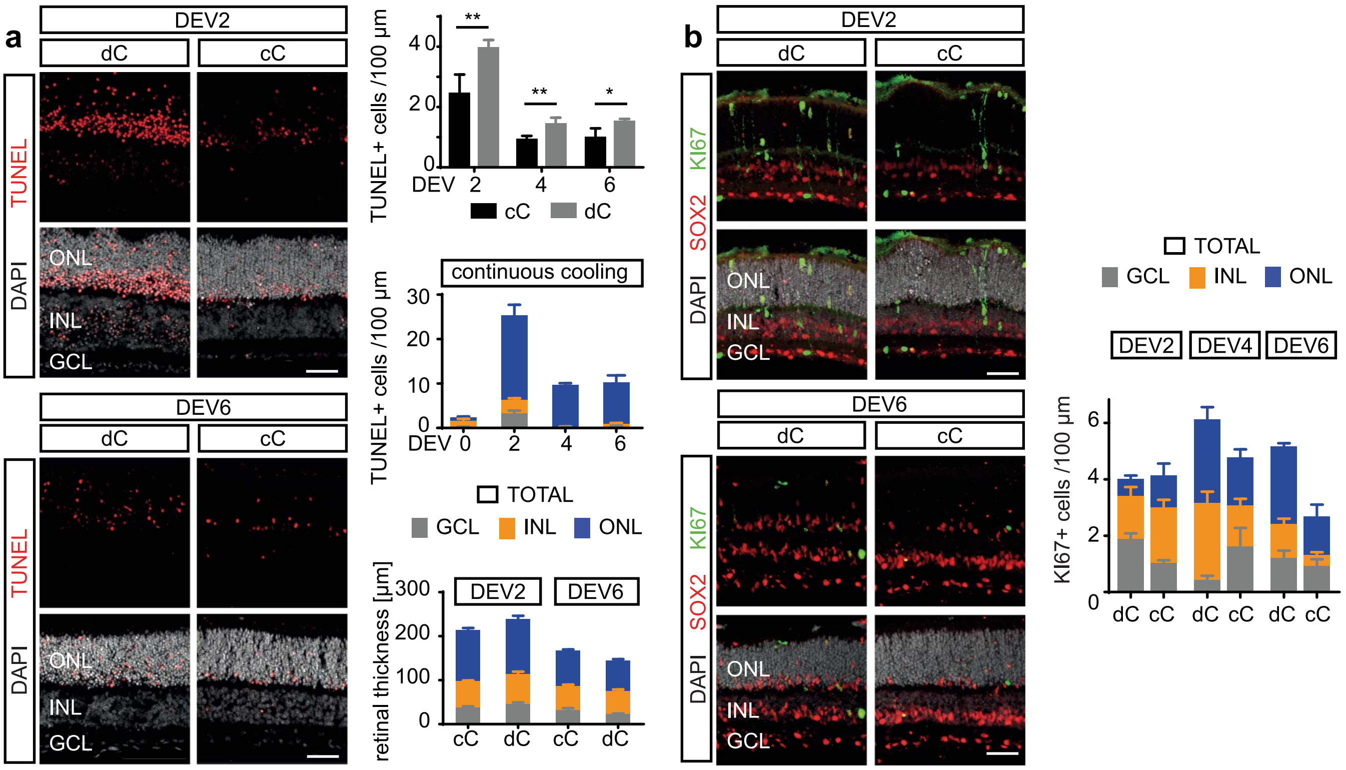

Supplement Figure S1: Supplement data for hypothermia pretreatment reduces neuron death and Müller glia proliferation.

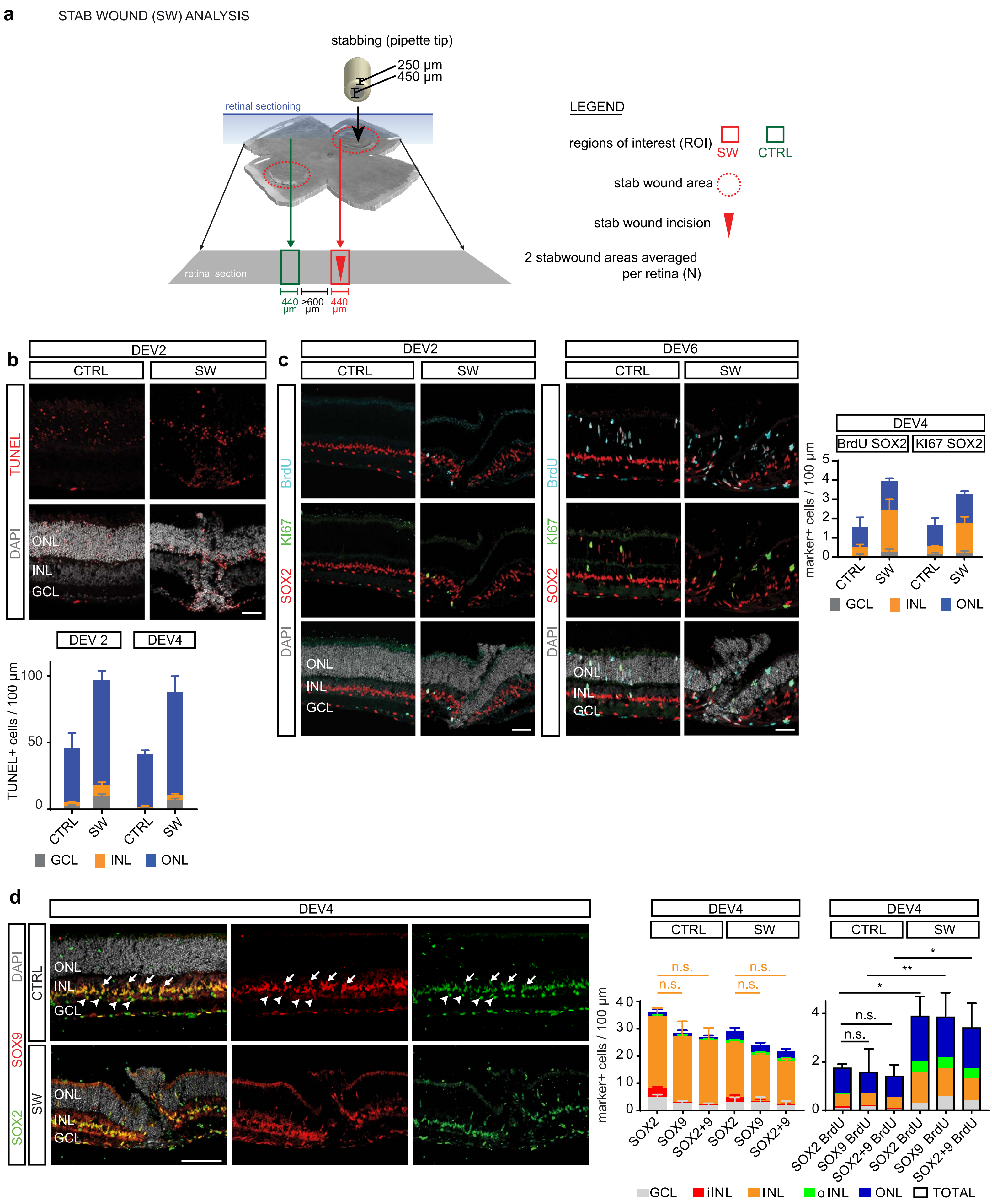

Supplement Figure S2: Supplement data for time course analysis of cell death and proliferation in retinal SW injury.

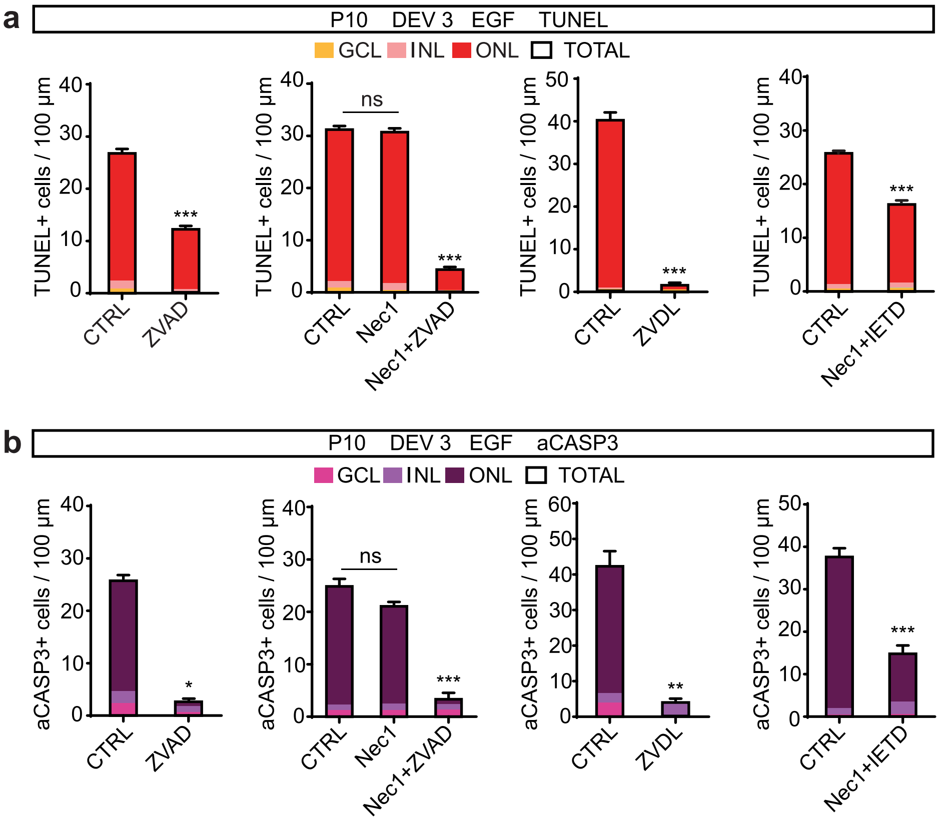

Supplement Figure S3: Supplement data for combined (not single) death pathway inhibition reduces retinal cell death.

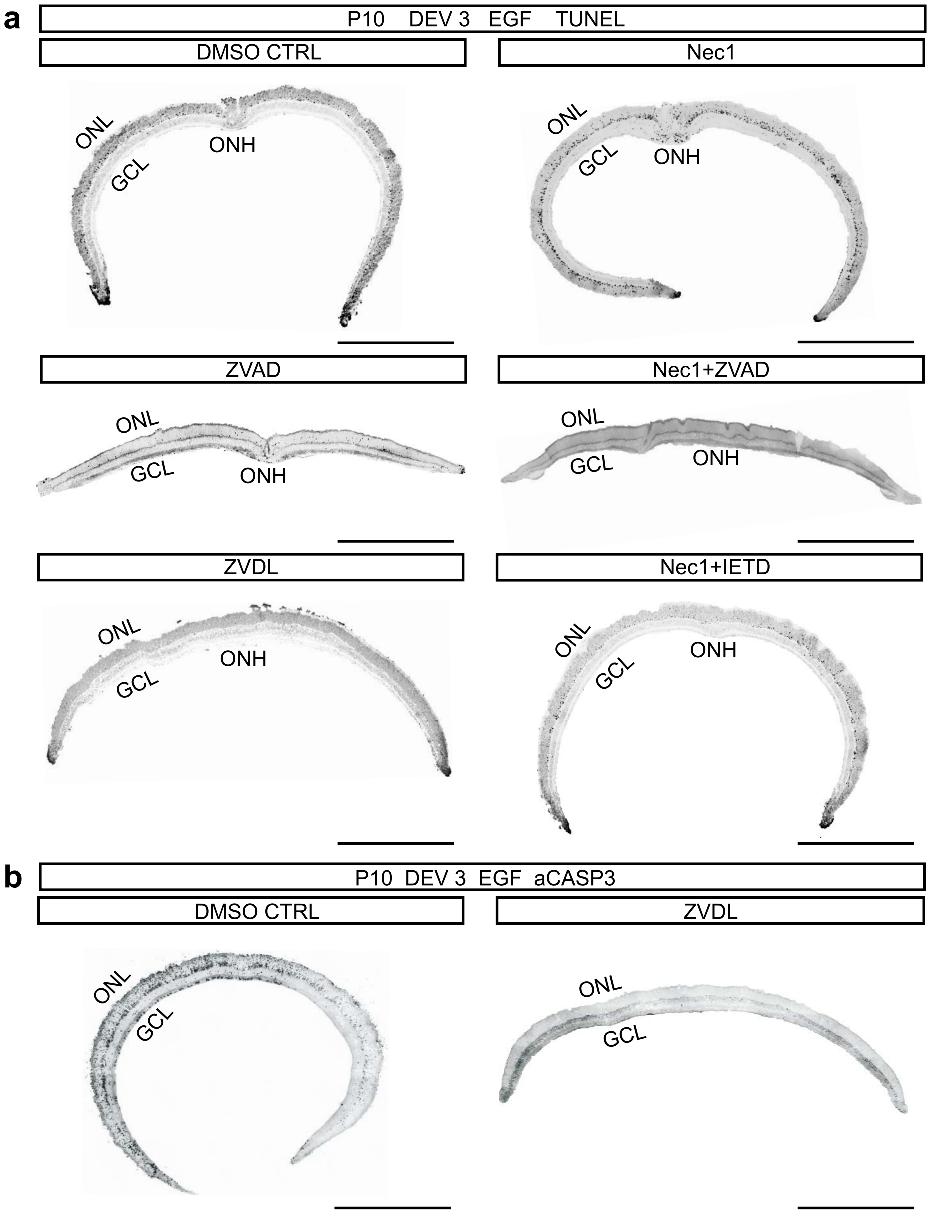

Supplement Figure S4: Supplement data for combined (not single) death pathway inhibition reduces retinal cell death.

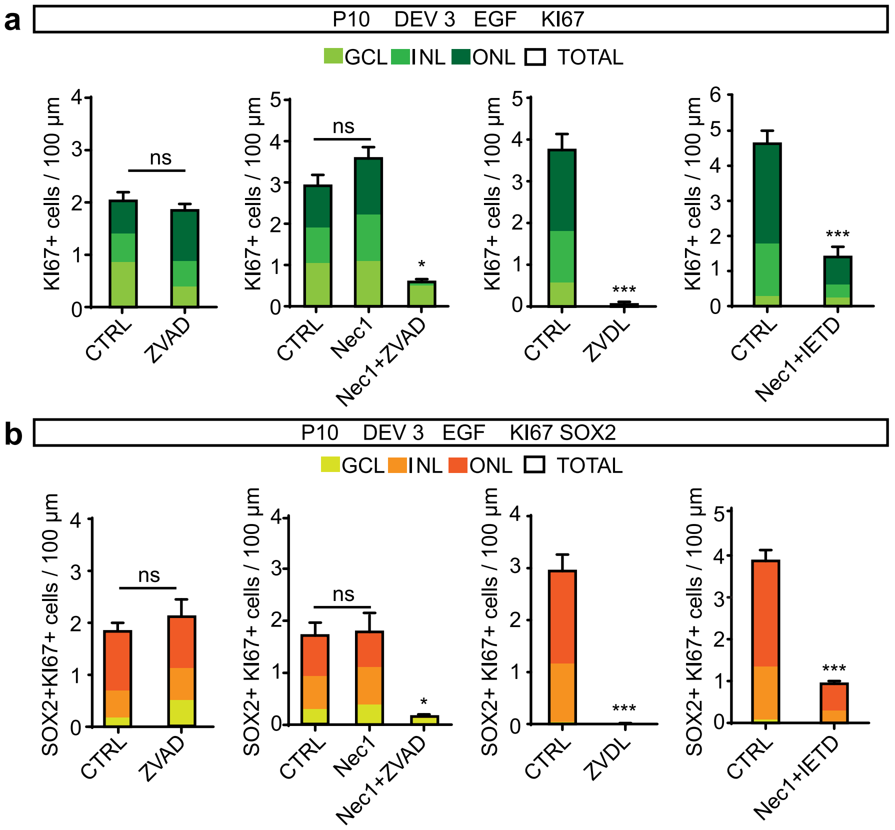

Supplement Figure S5: Supplement data for retinal cell death inhibition prevents Müller glia proliferation.

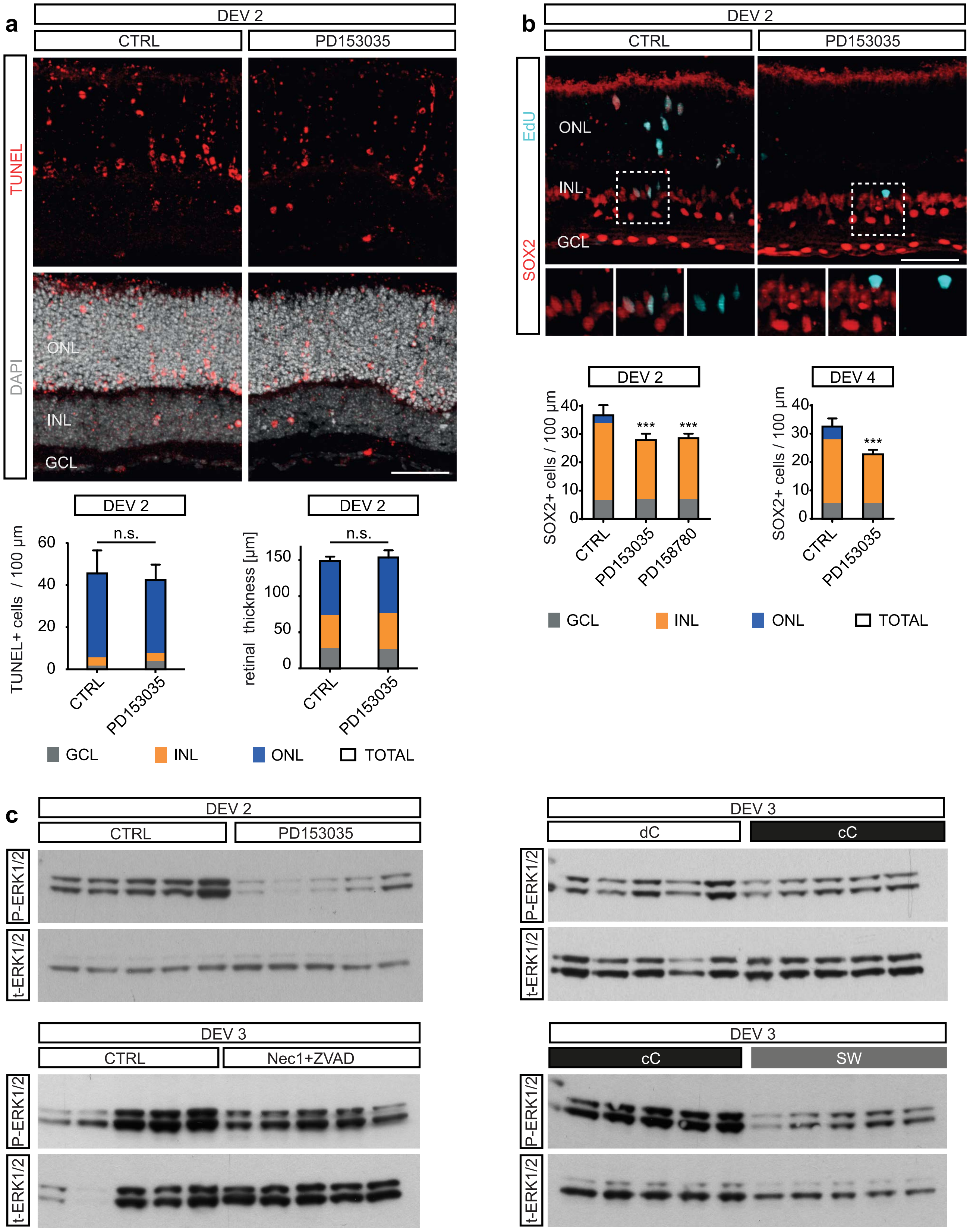

Supplement Figure S6: Supplemental data for EGFR–signaling is necessary for Müller glia proliferation and ERK1/2–activity and damage dependence of ERK1/2 activiation.

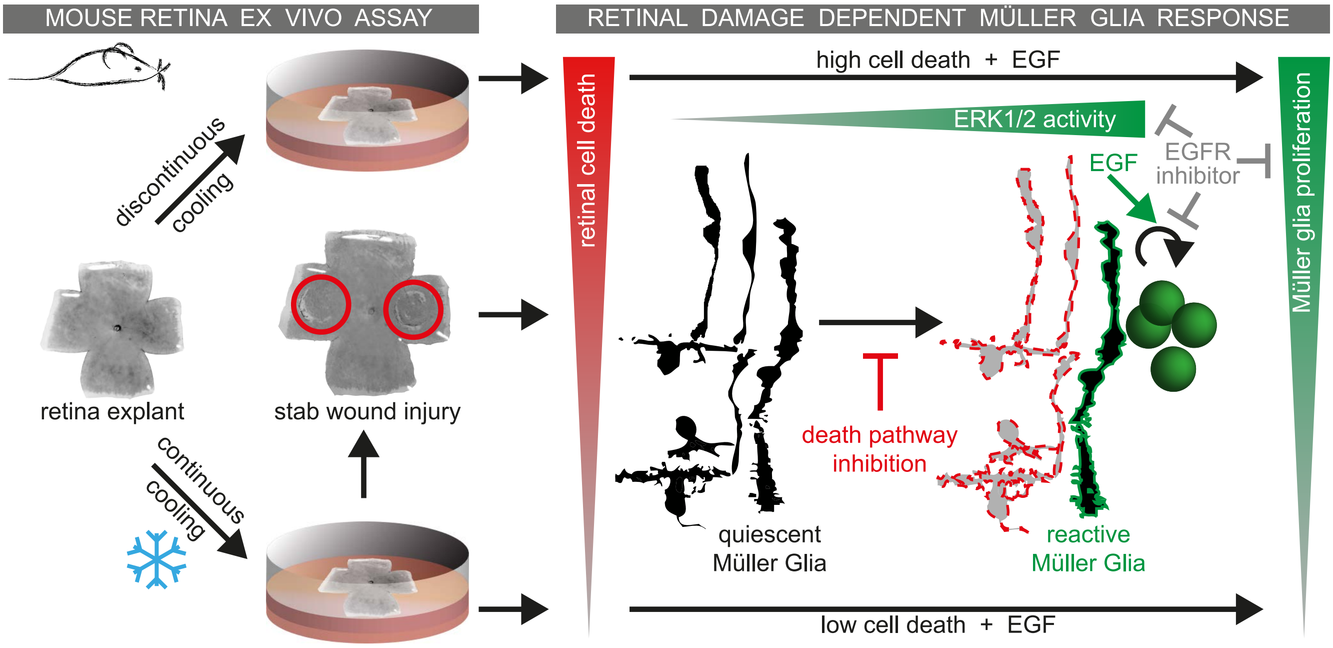

Supplement Figure S7: Supplement figure for summary of cell death–dependent Müller glia proliferation response in the mouse retina regeneration assay

Suppl. Table 1: Cell death pathway and EGFR Inhibitors

acronym	inhibitor name	source	catalogue number	final concentration	reported primary targets
NEC1	Necrostatin-1 (Nec1)	Calbiochem	480065	50 µM	RIPK1 and RIPK3
ZVAD	Caspase inhibitor VI (Z-VAD-FMK)	Calbiochem	219007	10 µM	broad spectrum caspase inhibitor
IETD	Caspase8 inhibitor II (Z-IETD-FMK)	Calbiochem	218759	10 µM	caspase 8 and granzyme B
ZVDL	Caspase inhibitor VI (Z-VAD-FMK)	Calbiochem	219007	10 µM	broad spectrum caspase inhibitor
	Caspase9 inhibitor I (Z-LEHD-FMK)	Calbiochem	218761	10 µM	caspase 4, 5 and 9
	Caspase3 inhibitor III (Z-DEVD-FMK)	Calbiochem	264155	10 µM	caspase 3, 6, 7, 8 and 10
PD153035	PD153035 hydrochloride	Tocris	1037	0.5 µM	epidermal growth factor receptor tyrosine kinase (EGFR)
PD158780	PD158780	Tocris	2615	0.5 µM	epidermal growth factor receptor tyrosine kinase (EGFR)

Suppl. Table 2: Antibody information

Primary Antibody	Immunogen	Marker	Source	Dilution, Species, Type
aCASP3	Human Active Caspase-3 Fragment.	cells undergoing apoptosis	BD 559565	1:500, rb, mc
aCASP3	Human Active Caspase-3 Fragment	cells undergoing apoptosis	Abnova, PAB8 619	1:100, rb, pc
TFAP2A	human AP-2 alpha delta N165	GABAergic AC	DSHB, 3B5	1:10, ms, mc
Anti-BrdU	recognizes BrdU incorporated into single stranded DNA	BrdU incorporated in S-phase of the cell cycle	AbD Serotec, (clone BU1/75)	1:250, rt, mc
KI67	A synthetic peptide from C-terminus of Human Ki-67.	Late G1-/S-/G2-/M-phase cell cycle	Acris, DRM004	1:200, rb, mc
KI67	Human Ki-67	Late G1-/S-/G2-/M-phase cell cycle	BD(clone B56, 550609)	1:500,ms, mc
P-ERK1/2	Anti-phospho-p44/42 (Erk1/2) Erk1 (Tyr204) /Erk2(Tyr187)	Mitogen-activated protein kinase (MAPK) cascade activation and detects dual-phosphorylated p44 MAPK/Erk1 (Thr202/Tyr204)/p42 MAPK/Erk2	Cell signaling (5726)	1:200, ms, mc
SOX2	peptide mapping near the C-terminus of Sox-2 of human origin	AS, AC, MG, RPC	Santa Cruz (Y-17) sc-17320	1:200, gt, pc
SOX9	Peptide mapping at C-terminus of SOX9 of human origin	AS, MG, RPC	Sigma-aldrich (HPA001758)	1:200, rb, pc

Table legend: AC, amacrine; AS, astrocyte; BP, bipolar; MG, Müller glia; PR, photoreceptor; RGC, retinal ganglion cell; RPC, retinal progenitor cell; pc, polyclonal; mc, monoclonal; rt, rat; ms, mouse; rb, rabbit; gt, goat.

Secondary Antibody	Host	Antigen	Source(catalog)	Dilution(Conc)
Alexa Fluor 488 goat (H+L)	donkey	goat	Dianova (705-545-147)	1:500(0.5mg/ml)
Alexa Fluor 488 mouse IgG (H+L)	donkey	mouse	Dianova (715-545-151)	1:500(0.5mg/ml)
Alexa Fluor 488 rabbit IgG (H+L)	donkey	rabbit	Dianova (711-545-152)	1:500(0.5mg/ml)
Alexa Fluor 488 rat IgG (H+L)	donkey	rat	Dianova (712-545-153)	1:500(0.5mg/ml)
Indocarbocyanin Cy3 goat IgG (H+L)	donkey	goat	Dianova (705-165-147)	1:500(0.5mg/ml)
Indocarbocyanin Cy3 mouse IgG (H+L)	donkey	mouse	Dianova (715-165-151)	1:500(0.5mg/ml)
Indocarbocyanin Cy3 rat IgG (H+L)	donkey	rabbit	Dianova (712-165-153)	1:500(0.5mg/ml)
Indocarbocyanin Cy3 rabbit IgG (H+L)	donkey	rat	Dianova (711-165-152)	1:500(0.5mg/ml)
Alexa Fluor 647 goat IgG (H+L)	donkey	goat	Dianova (705-605-147)	1:500(0.5mg/ml)
Alexa Fluor 647 mouse IgG (H+L)	donkey	mouse	Dianova (715-605-151)	1:500(0.5mg/ml)
Alexa Fluor 647 rabbit IgG (H+L)	donkey	rabbit	Dianova (711-605-152)	1:500(0.5mg/ml)
Alexa Fluor 647 rat IgG (H+L)	donkey	rat	Dianova (712-605-153)	1:500(0.5mg/ml)

Suppl. Table 3: QPCR primers

Gene	Primer	5´– 3´sequence	length (bp)	amplicon size (bp)	Source
Actb	Fwd	CTAAGGCCAACCGTGAAAAG	20	104	La Torre et al., 2012
	Rev	ACCAGAGGCATACAGGGACA	20		
Ripk1	Fwd	CTTGCTGTCATCTAGCGGGA	20	76	designed, Primer-blast
	Rev	GTGCTGTGTCCTTCAGTCTCG	21		
Ripk3	Fwd	GCCTTCCTCTCAGTCCACAC	20	127	designed, Primer-blast
	Rev	ACGCACCAGTAGGCCATAAC	20		
Casp3	Fwd	TACTCTACAGCACCTGGTTAC	21	148	Gehrig et al., 2006
	Rev	CCGTTGCCACCTTCCTGTT	19		
Casp7	Fwd	CGGAATGGGACGGACAAAGA	20	135	Gehrig et al., 2006
	Rev	GTGGTCCTCCTCAGAGGCTT	20		
Casp8	Fwd	AAGATGTCCTCAAGGAGATG	20	91	Gehrig et al., 2006
	Rev	CTTTCCCTTGTTCCTCCTGT	20		

References

La Torre A, Lamba DA, Jayabalu A, Reh TA. 2012. Production and transplantation of retinal cells from human and mouse embryonic stem cells. Methods Mol Biol 884:229-46.

Gehrig A, Janssen A, Horling F, Grimm C, Weber BH. 2006. The role of caspases in photoreceptor cell death of the retinoschisin-deficient mouse. Cytogenet Genome Res 115(1):35-44.

Suppl. Table 4: Summary of experimental datasets and biological replicates (N)

Figure	Panel	associated supplement figure	Panel	Datasets	Variable	N
1	c, e, g			Temporal response dC: TUNEL, aCASP3, KI67	DEV 0	4
					DEV 0.65	3
					DEV 1	4
					DEV 2	3
					DEV 3	3
					DEV 4	4
					DEV 6	3
2	b	S1	a	cC vs dC: TUNEL, thickness	dC DEV 2	3
					dC DEV 4	4
					dC DEV 6	3
					cC DEV 2	4
					cC DEV 4	4
					cC DEV 6	4
2	c	S1	b	cC vs dC: KI67	dC DEV 2	4
					dC DEV 4	4
					dC DEV 6	4
					cC DEV 2	4
					cC DEV 4	4
					cC DEV 6	4
3	b	S2	b	Stab wound: TUNEL	CTRL DEV 2	3
					CTRL DEV 4	4
					SW DEV 2	3
					SW DEV 4	4
3	c	S2	c	Stab wound: KI67, SOX2+KI67+, BrdU+KI67+	CTRL DEV 2	4
					CTRL DEV 4	4
					SW DEV 2	4
					SW DEV 4	4
3		S2	d	Stab wound: SOX2 vs SOX9 comparison	CTRL DEV 4	4
					SW DEV 4	4
4	b, d	S3	a, b	Cell death inhibition: TUNEL, aCASP3	CTRL for Nec1 and Nec1+ZVAD	4
					CTRL for ZVAD	4
					CTRL for ZVDL	3
					CTRL for Nec1+IETD	4
					Nec1	4
					ZVAD	4
					Nec1+ZVAD	4
					ZVDL	4
					Nec1+IETD	4
4	f			Temporal response: cell death qPCR	DEV 0	4
					DEV 2	4
					DEV 4	4
					DEV 7	4
4	g			Cell death inhibition: cell death qPCR	CTRL for ZVAD and Nec1	4
					CTRL for ZVDL and Nec1+ZVAD	3
					ZVAD	4
					NEC-1	4
					Nec1+ZVAD	4
					ZVDL	4

Figure	Panel	associated supplement figure	Panel	Datasets	Variable	N
5	b, d	S5	a, b	Cell death inhibition: KI67, KI67+SOX2+	CTRL for Nec1 and Nec1+ZVAD	4
					CTRL for ZVAD	4
					CTRL for ZVDL	3
					CTRL for Nec1+IETD	4
					Nec1	4
					ZVAD	4
					Nec1+ZVAD	4
					ZVDL	4
					Nec1+IETD	4
6	a			Temporal response: P-ERK1/2+SOX2+	DEV 0	7
					dC DEV 0.75	5
					dC DEV 2	7
					dC DEV 4	4
6	b, c	S6	b	EGFR inhibition: EdU+SOX2+	CTRL DEV 2	4
					CTRL DEV 4	5
					PD153035 DEV 2	4
					PD158780 DEV 2	4
					PD153035 DEV 4	5
					PD158780 DEV 4	3
6	d			EGFR inhibition: P-ERK1/2-WB	CTRL DEV 2	5
					PD153035 DEV 2	5
6		S6	a	EGFR inhibition: TUNEL, thickness	CTRL	3
					PD153035	4
7	a			cC vs dC: P-ERK1/2+SOX2+	dC DEV 2	7
					dC DEV 4	9
					cC DEV 2	7
					cC DEV 4	9
7	a			Stab wound: P-ERK1/2+SOX2+	CTRL DEV 2	4
					CTRL DEV 4	4
					SW DEV 2	4
					SW DEV 4	4
7	b			Cell death inhibition: P-ERK1/2+SOX2+	CTRL Nec1+ZVAD	8
					CTRL ZVDL	4
					CTRL Nec1+IETD	4
					Nec1+ZVAD	8
					ZVDL	4
					Nec1+IETD	4
7	c			Damage dependence: P-ERK1/2-WB	CTRL dC DEV 3	5
					CTRL cC for SW DEV 3	5
					CTRL DMSO for Nec+ZVAD	5
					cC DEV 3	5
					SW DEV 3	5
					Nec1+ZVAD DEV 3	5

Table legend: N, biological replicates; DEV, days ex vivo; dC, discontinuous cooling; cC, continuous cooling; CTRL, control (solvent or unlesioned); SW, stab wound; WB, Western blot
